# A Germin-Like Protein GLP1 of Legumes Mediates Symbiotic Nodulation by Interacting with an Outer Membrane Protein of Rhizobia

**DOI:** 10.1128/spectrum.03350-22

**Published:** 2023-01-12

**Authors:** Xiaobo Zeng, Dongzhi Li, Yanfei Lv, Yao Lu, Lingli Mei, Donglai Zhou, Dasong Chen, Fuli Xie, Hui Lin, Youguo Li

**Affiliations:** a State Key Laboratory of Agricultural Microbiology, College of Life Science and Technology, Huazhong Agricultural University, Wuhan, People’s Republic of China; USDA—San Joaquin Valley Agricultural Sciences Center

**Keywords:** outer membrane proteins, OMPs, germin-like protein, interaction, rhizobium, legume, early symbiosis, RNA-seq

## Abstract

Rhizobia can infect legumes and induce the coordinated expression of symbiosis and defense genes for the establishment of mutualistic symbiosis. Numerous studies have elucidated the molecular interactions between rhizobia and host plants, which are associated with Nod factor, exopolysaccharide, and T3SS effector proteins. However, there have been relatively few reports about how the host plant recognizes the outer membrane proteins (OMPs) of rhizobia to mediate symbiotic nodulation. In our previous work, a gene (*Mhopa22*) encoding an OMP was identified in Mesorhizobium huakuii 7653R, whose homologous genes are widely distributed in *Rhizobiales*. In this study, a germin-like protein GLP1 interacting with Mhopa22 was identified in *Astragalus sinicus*. RNA interference of AsGLP1 resulted in a decrease in nodule number, whereas overexpression of AsGLP1 increased the number of nodules in the hairy roots of *A. sinicus*. Consistent symbiotic phenotypes were identified in Medicago truncatula with *MtGLPx* (refer to *medtr7g111240.1*, the isogeny *of AsGLP1*) overexpression or Tnt1 mutant (*glpx-1*) in symbiosis with Sinorhizobium meliloti 1021. The *glpx-1* mutant displayed hyperinfection and the formation of more infection threads but a decrease in root nodules. RNA sequencing analysis showed that many differentially expressed genes were involved in hormone signaling and symbiosis. Taken together, AsGLP1 and its homology play an essential role in mediating the early symbiotic process through interacting with the OMPs of rhizobia.

**IMPORTANCE** This study is the first report to characterize a legume host plant protein to sense and interact with an outer membrane protein (OMP) of rhizobia. It can be speculated that GLP1 plays an essential role to mediate early symbiotic process through interacting with OMPs of rhizobia. The results provide deeper understanding and novel insights into the molecular interactive mechanism of a legume symbiosis signaling pathway in recognition with rhizobial OMPs. Our findings may also provide a new perspective to improve the symbiotic compatibility and nodulation of legume.

## INTRODUCTION

Legumes, as an important source of food, feed and biofuel, can establish a complex symbiotic relationship with specific soil bacteria collectively called rhizobia. This symbiosis will result in the formation of a special plant structure called nodules on the roots and stems of the host plant. Within the nodules, rhizobia will differentiate into bacteroids, which can fix atmospheric nitrogen into ammonia to be used by the host plant. In return, rhizobia can obtain reduced carbon and other nutrients from the host plant (1). It has been well documented that the establishment of symbiosis between legumes and compatible rhizobia depends on mutual signal perception, interaction, and regulation (2).

Multiple nodulation strategies have been identified, particularly the Nod factor (NF)-dependent strategy (3). NFs, lipochitooligosaccharides of rhizobia, are necessary and sufficient to induce nodule development on legume roots, even in the absence of rhizobia (4). Further investigation revealed that NFs are recognized by specific NF receptors, a kind of plant-plasma-membrane-localized serine/threonine receptor kinase (5), such as *MtLYK3*/*MtLYK4* and *MtNFP* in *M. truncatula* (6–8), and *LjNFR1* and *LjNFR5* in *L. japonicus* (9), which are crucial for triggering the Nod-dependent signaling pathway. In addition to NFs, the interaction between bacterial surface exopolysaccharides (EPS) and plant plasma membrane receptors was recently identified. The EPS receptor LjEPR3 in *L. japonicus*, a LysM domain-containing entry receptor, is dispensable for early response to NFs but essential for infection progression (10). Furthermore, other diverse polysaccharides on the surfaces of rhizobia, such as KPS (K polysaccharides) and LPS (lipopolysaccharides), also play important roles in the process of symbiosis establishment (11). Although the molecular mechanism remains to be revealed, it is believed that NFs, LPS, and EPS are lipid oligosaccharides or lipid-linked oligosaccharides, and the lengths of glycan, types, and degrees of functional group modification allow the specific recognition between rhizobia and host plants (2, 5, 12).

On the other hand, there are various microbe-associated molecular patterns (MAMPs) in Gram-negative bacteria, such as flagellin (Flg), elongation factor Tu (EF-Tu), and peptidoglycan (PGN). These MAMPs are recognized by plant surface-localized pattern recognition receptors (PRRs) (13), which can elicit MTI (MAMP-triggered immunity) to enhance resistance to microbial invasion in roots (14, 15). However, the rhizobial MAMPs identified so far (including flagellin, LPS, and PGN) do not trigger MTI in their hosts (16–18). Based on the recognition between MAMPs and PRRs, the roots distinguish “friend or foe,” recognizing beneficial microbes and pathogenic ones. Beneficial microbes have to evade the host plant immune response or modify the recognition by the host plant receptors in order to establish root symbiosis interactions.

Apart from lipids and polysaccharides, abundant and diverse outer membrane proteins (OMPs) are integrated or embedded in the outer membrane of Gram-negative bacteria. It has been reported that there are at least 45 kinds of OMPs in Escherichia coli (19). Structurally, integral OMPs generally possess β-barrel arranged with 8 to 26 antiparallel beta folds (20). The transmembrane domain or extracellular part of OMPs confers diverse functions, including nutrient absorption, cell adhesion, signal transduction, and infection. Inactivation of the *Omp25* gene of Brucella resulted in a significant decrease in virulence relative to the wild type (21) because Omp25 can induce the secretion of inflammatory cytokines by microglial cells and inhibit apoptosis, thereby enhancing their colonization ability in the host (22). Anaplasma marginale outer membrane protein A (*AmOmpA*) is a kind of adhesin that can interact with sialylated and fucosylated glycan receptor in host cells, which mediates its invasion into bovine blood cells (23). In Xanthomonas axonopodis pv. glycines, the OmpA-mediated invasion to soybean plays an important role in protein secretion during pathogenesis due to significant decreases in the production of cellulase, pectate lyase, and polysaccharide and in pustule number observed with the *ompA* mutant compared to the wild type (24).

However, very few studies have characterized the recognition or interaction between OMPs and host plant cells, especially in the process of symbiotic nodulation and nitrogen fixation, though a few OMPs have been identified in rhizobia. The adhesion proteins RapA, RapB, and RapC identified in R. leguminosarum bv. trifolii are involved in the biofilm formation that facilitates the colonization of R. leguminosarum on the root of the host plant (25, 26) but have no effect on nodulation (27). The FadL_Sm_ OMP in S. meliloti is a fatty acid transporter that facilitates the diffusion of long-chain N-acyl homoserine lactones (28), which in turn plays an essential role in quorum sensing (29), especially in the process of symbiotic establishment between rhizobia and legumes (30). In Rhizobium etli CFN42, *ropAe* encoding an OMP conferred the function of regulating the Cu^2+^ concentration (31). However, the function mechanisms of both *FadL_Sm_* and *ropAe* in symbiosis are largely unknown. The RopB of *Rhizobiales* was found to play an important role in maintaining outer membrane stability (32, 33) and may participate in the long-chain fatty acid modification of lipid A together with *acpXL* (34). Recently, S. meliloti outer membrane protein NsrA was identified to transduce *Medicago* host plant signals to adenylate cyclases in the inner membrane, thereby triggering a cAMP signaling cascade that controls infection (35).

The symbiosis between *M. huakuii* 7653R and *A. sinicus* has been studied extensively and applied in sustainable agriculture for many years (36, 37), and some symbiosis-related genes of *M. huakuii* 7653R have been identified, such as *lpsH* (38), *hfq* (39), and *htpG* (40). It is worth noting that Opa22 (renamed Mhopa22 here) is a novel membrane protein required for nodulation identified in *M. huakuii* 7653R. Phylogenetic analyses have shown that *Mhopa22* and its homologous genes are widely distributed in *Rhizobiales* (41, 42). Here, we screened and identified a germin-like protein (AsGLP1) from *A. sinicus* by using Mhopa22 as bait via the yeast two-hybrid (Y2H) system. Further experiments confirmed the interaction between Mhopa22 and AsGLP1, and the role of *GLP1* in nodule symbiosis was characterized in the *A. sinicus*-*M. huakuii* 7653R and *M. truncatula*-S. meliloti 1021 systems. The results provide novel insights into the molecular mechanism for the interaction between the symbiosis signaling pathway of legumes and rhizobial OMPs.

## RESULTS

### Identification of the interaction partner of Mhopa22 in the host plant.

Mhopa22 is a mesorhizobial OMP required for nodulation and has a conserved Surface_Ag_2 domain and a conserved β-barrel structure (42). In order to identify the host plant protein interacting with Mhopa22, the Y2H screening was performed with an *A. sinicus* cDNA library using Mhopa22 as a bait protein. As a result, six cDNA clones encoding candidate interacting proteins were obtained (see Table S1 in the supplemental material), and the interactions between those captures and Mhopa22 were checked by filter paper print (see Fig. S1).

**FIG 1:**
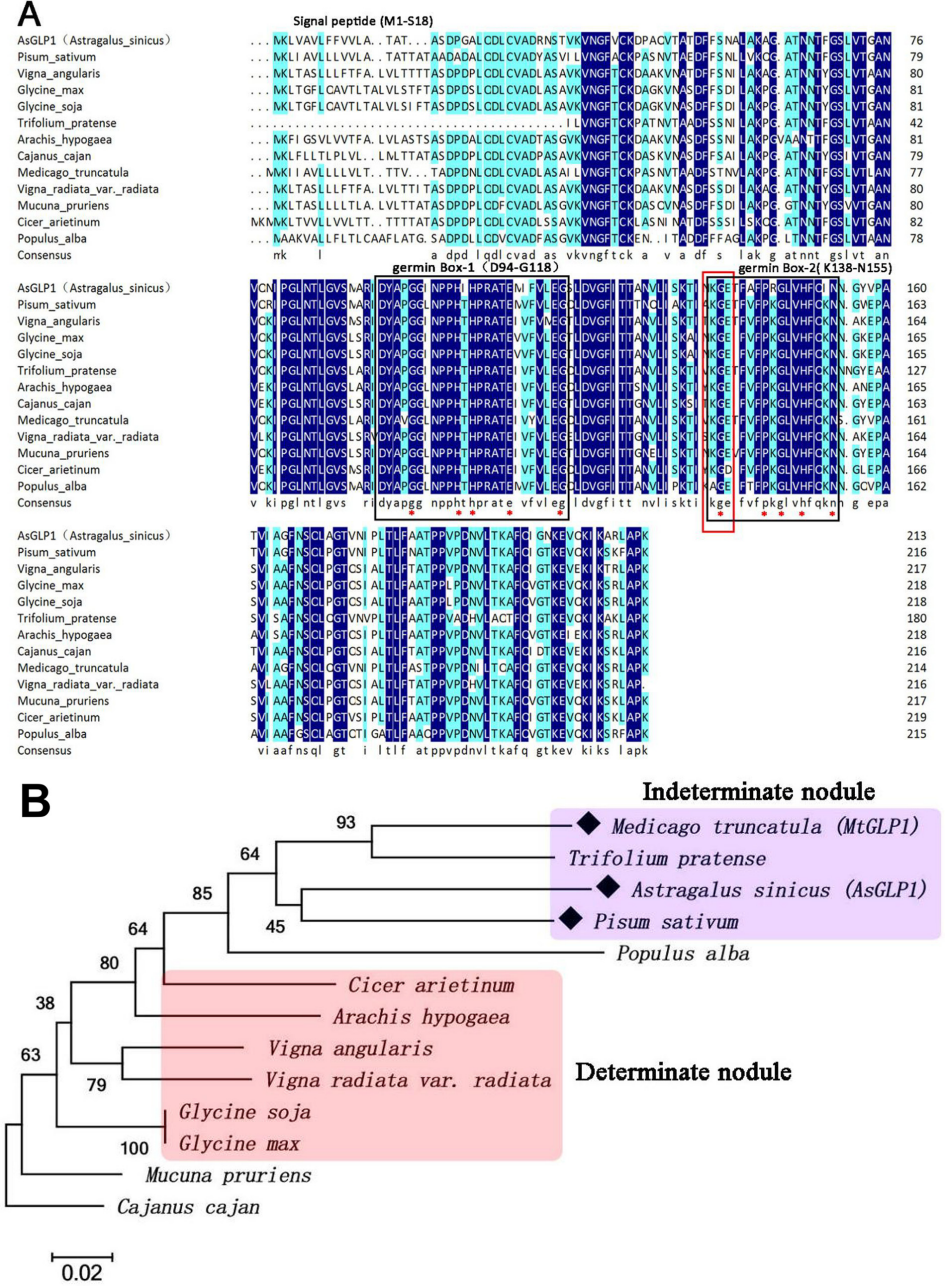
Phylogenetic analysis of AsGLP1 homologous proteins in various leguminosae. (A) Amino acid sequence alignment of AsGLP1 and its orthologs. Amino acids forming the germin boxes are indicated by black solid squares (their scope is indicated via abbreviation and location of amino acid residue in brackets), and the red asterisks indicate conserved residues. The red rectangle indicates tripeptide KGD (or sometimes KGE). The 18 amino acid residues in N-terminal predicted signal peptide are indicated. (B) Phylogenetic tree of AsGLP1 and its orthologs constructed by the neighbor-joining method. The amino acid sequence of AsGLP1 homologous proteins annotated as rhicadhesin receptor or rhicadhesin receptor-like was obtained from NCBI data. Medicago truncatula (XP_003626120.1), Trifolium pratense (PNX83803.1), Pisum sativum (Q9S8P4.2), *Populus alba* (TKS04171.1), *Cicer arietinum* (XP_004494499.1), Arachis hypogaea (XP_025662292.1), *Vigna angularis* (XP_017417070.1), *Vigna radiata* var. radiata (XP_014495130.1), *Glycine soja* (XP_028218134.1), Glycine max (XP_003554594.1), *Mucuna pruriens* (RDX74394.1), and Cajanus cajan (XP_020210822.1).

Interestingly, one of the candidate proteins screened in the clone Pro-13 and annotated as “rhicadhesin receptor” was found to play an important role in mediating the direct attachment of bacteria to plant cell surface (43). Since the establishment of symbiosis involves the recognition of signaling molecules between rhizobia and host plants, we focused on the interaction verification and functional characterization of this gene. Given that there was no available genomic sequence for *A. sinicus*, we obtained the complete coding sequence (CDS) of this protein from our RNA sequencing (RNA-seq) data (unpublished) and submitted it to the NCBI database under accession number MZ669228. The predicted protein encoded by this CDS was composed of 213 amino acids with a molecular weight of 22.5 kDa and belonged to germin-like proteins (a subclass of germination protein superfamily). Therefore, we designated this gene *AsGLP1* in *A. sinicus*. The bioinformatic analysis predicted that the AsGLP1 protein sequence contains an N-terminal signal peptide of 18 amino acids, two highly conserved germin boxes (germin box 1 and germin box 2) for the formation of a cupin domain and a putatively important sequence KGE located in germin box 2 (Fig. 1A). BLASTp analysis revealed that AsGLP1 homologous proteins are present in diverse leguminous plants and share more than >70% sequence identities among *Vigna angularis*, Glycine max, Cajanus cajan, and *M. truncatula* (Fig. 1A). A phylogenetic tree was further constructed with the neighbor-joining method in MEGA 6. Analysis revealed that GLP1 homologs were widely distributed in legumes that form indeterminate and determinate nodules (Fig. 1B) and showed that GLP1 is conserved in different legumes and may play different roles in the formation and development of the two types of nodules.

### Verification of protein interaction between AsGLP1 and Mhopa22 *in planta* and *in vitro*.

**FIG 2:**
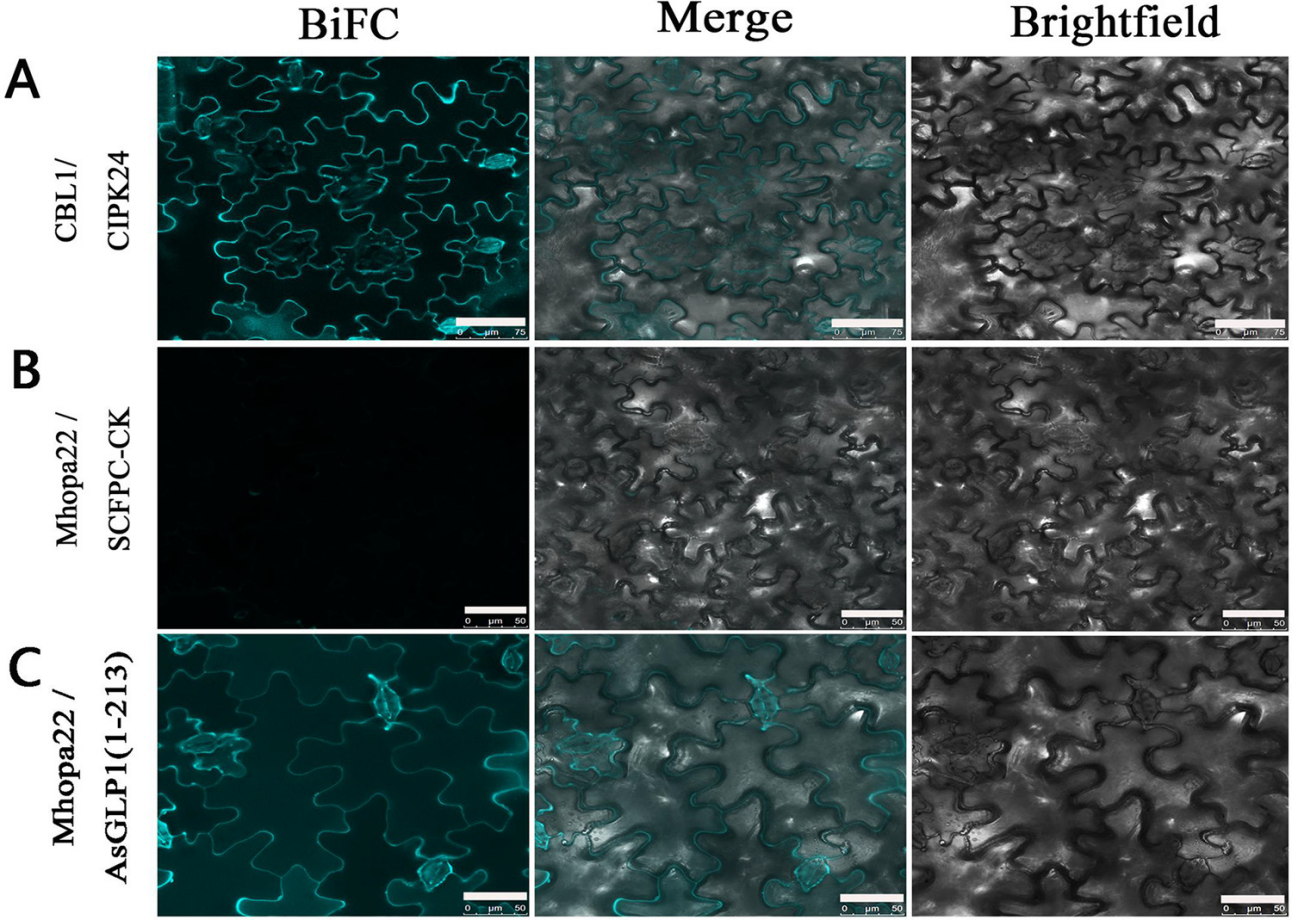
Identification of the protein interaction between Mhopa22 and AsGLP1 in tobacco leaf cells by BiFC. Various pairs were cotransformed into N. benthamiana leaves. (A) CBL1/CIPK24 represents the cotransformation of SCFPN::CBL1 with CIPK24::SCFPC as the positive control. (B) Mhopa22/SCFPC-CK represents the cotransformation of SCFPN::Mhopa22 with SCFPC (the original plasmid without inserted fragment) as the negative control. (C) Mhopa22/AsGLP1(1-213) represents the cotransformation of Mhopa22 with AsGLP1(1-213)::SCFPC. CFP fluorescence was observed in tobacco leaf epidermal cells using a confocal laser microscope. Because the CFP was split into N- and C-terminal halves and fused with different proteins, no cyan fluorescence would be observed if the fusion proteins did not interact. As a positive control, CIPK24 and (CBL1) were, respectively, fused with the C-terminal CFPC155 and N-terminal CFPN173 and coexpressed. Scale bars, 75 μm (panel A) and 50 μm (panels B and C). The results confirm the interaction between Mhopa22 and full-length AsGLP1.

In order to validate the interaction between AsGLP1 and Mhopa22, bimolecular fluorescence complementation (BiFC) assays *in planta* were performed. The results showed that in the cotransformed tobacco leaves, strong cyan fluorescence signals were detected in the SCFPN::CBL1/CIPK24::SCFPC groups that were set as the positive control (Fig. 2A) and in SCFPN::Mhopa22/AsGLP1(1-213)::SCFPC (Fig. 2C). Meanwhile, there was no fluorescence signal in SCFPN::Mhopa22/SCFPC that was set as the negative control (Fig. 2B). These results indicated that full-length AsGLP1 interacts with Mhopa22.

**FIG 3:**
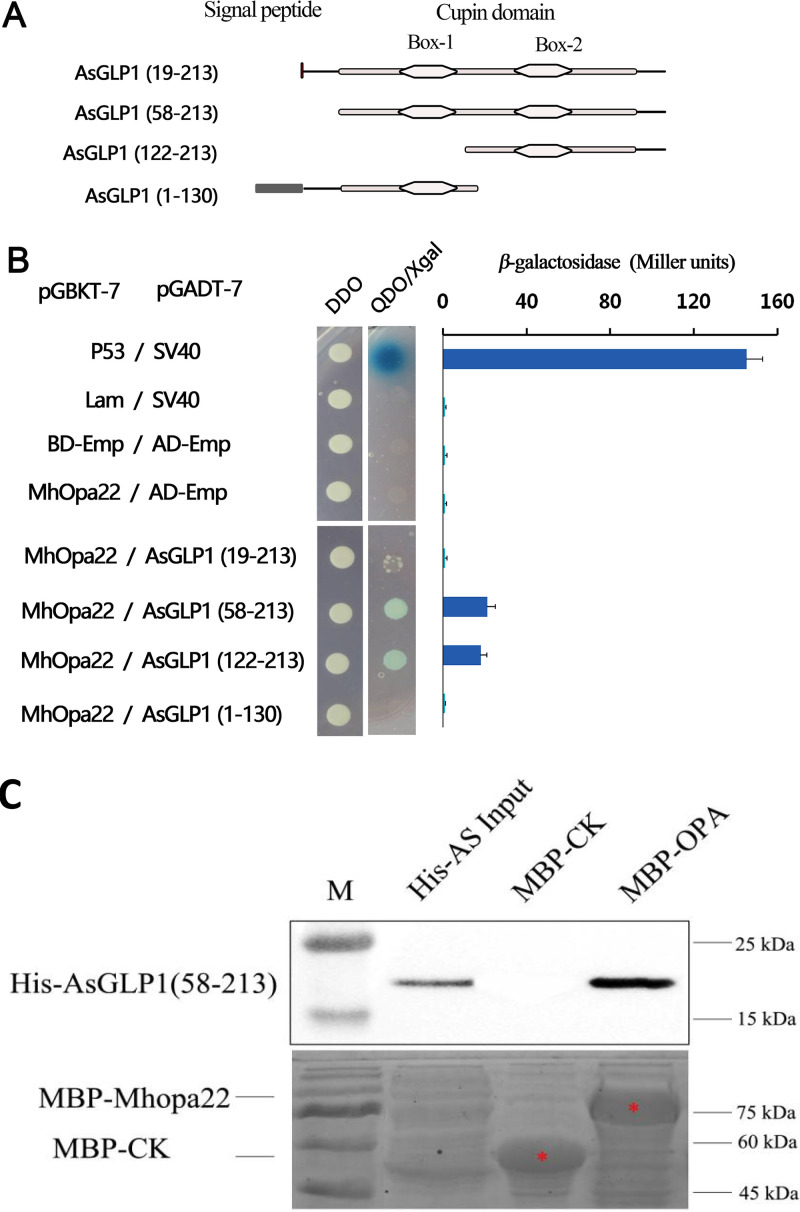
Verification of the interaction between Mhopa22 and AsGLP1 regions. (A) Schematic diagrams of the functional domains and four truncated constructs of AsGLP1. AsGLP1 contains a signal peptide and a cupin domain. The names assigned to the truncations are listed on the left of the panel, and the reserved amino acid residues are also shown in brackets. (B) Two-hybrid assays for the interactions between Mhopa22 and AsGLP1 different truncations. Combinations of BD-Emp/AD-Emp, Mhopa22/AD-Emp, and lam/SV40 served as negative controls, and p53/SV40 was the positive control. The interaction was strong, as indicated by β-galactosidase activity assay. DDO, SD/–Trp/–Leu; QDO-Xgal, SD/–Trp/Leu/–His/–Ade containing X-Gal (5-bromo-4-chloro-3-indolyl-β-d-galactopyranoside). (C) Pulldown assay of protein-protein interaction between Mhopa22 and AsGLP1(58-213). The positions of His-AsGLP1(58-213), MBP-Mhopa22 and MBP are indicated. Asterisks indicate the expression of MBP-Mhopa22 and MBP.

Furthermore, a Y2H assay was performed to identify the interaction between Mhopa22 and various truncated forms of AsGLP1 (Fig. 3A). All the yeast cells containing both bait and prey plasmids (original or derivative) could grow on the SD/–Trp/–Leu (DDO) agar plate, while on the SD/–Trp/Leu/–His/–Ade agar plate containing X-Gal (5-bromo-4-chloro-3-indolyl-β-d-galactopyranoside) (QDO/X), only the yeast cells containing BD-Mhopa22/AD-AsGLP1(58-213) or BD-Mhopa22/AD-AsGLP1(122-213) could form normal colonies, but those carrying either BD-Mhopa22/AD-AsGLP1(1-130) or BD-Mhopa22/AD-AsGLP1(19-213) could not (Fig. 3B). The yeast cells cotransformed with either BD or AD empty vectors did not form colonies, indicating that the fragments contained in BD or AD had no self-activating activity. The results suggested that AsGLP1(58-213) is essential for the interaction between AsGLP1 and Mhopa22.

To confirm the interaction between truncated AsGLP1(58-213) and Mhopa22, *in vitro* pulldown assay was performed. The results showed that AsGLP1(58-213)-6×His could be captured by MBP-Mhopa22(J) (Mhopa22 without signal peptide), but not by MBP-CK (Fig. 3C). Taken together, the results showed that a cupin box domain located at the C terminus of AsGLP1 is critical for mediating its interaction with Mhopa22.

These results confirmed the protein-protein interactions between Mhopa22 and AsGLP1 *in planta* and *in vitro*, and the crucial region of AsGLP1 to mediate its interaction with Mhopa22 was located at the C terminus containing the intact cupin box domain.

### Temporal and spatial expression patterns of *AsGLP1* and *Mhopa22*.

**FIG 4:**
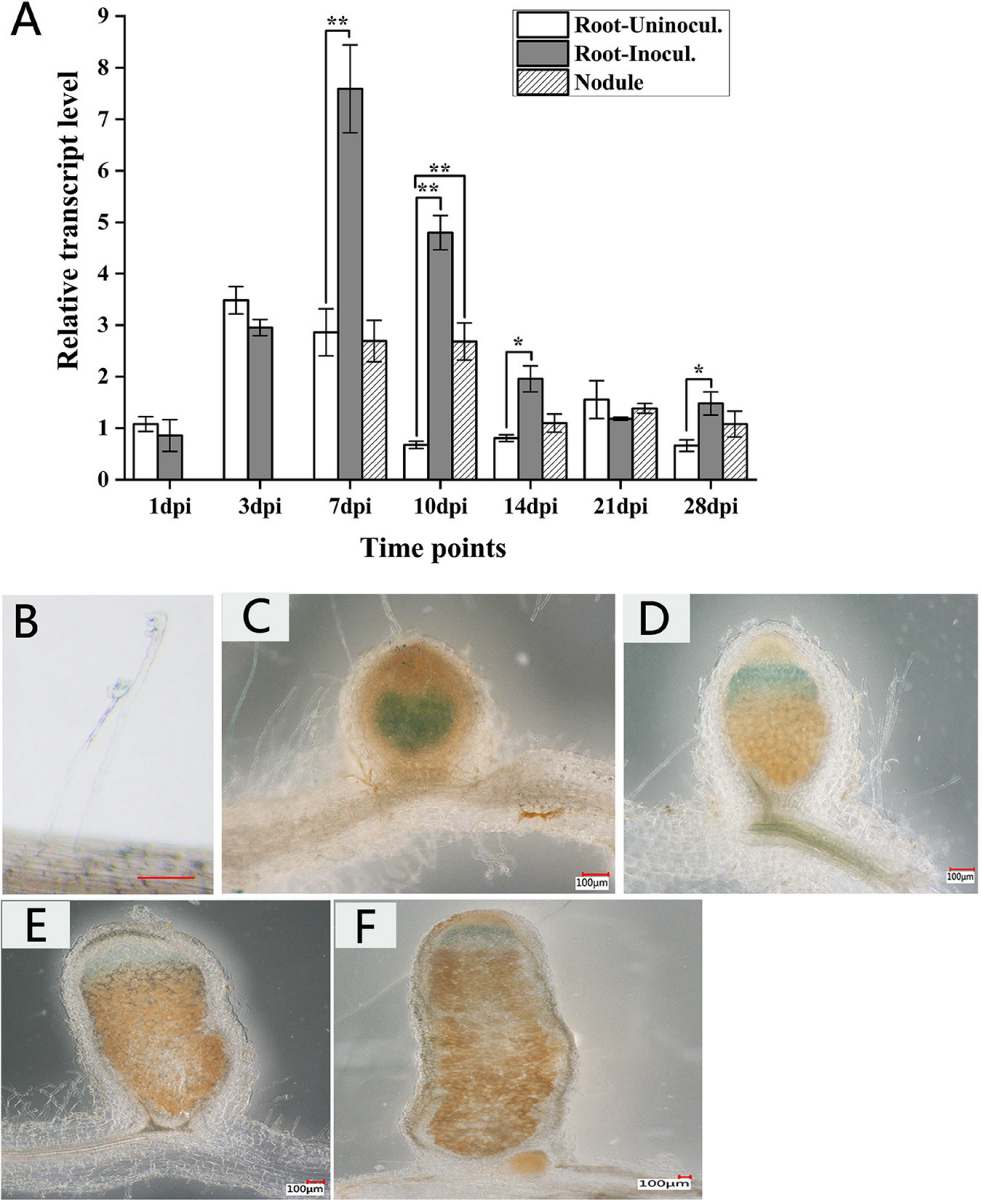
Temporal and spatial expression patterns of *AsGLP1* and *Mhopa22*. (A) Expression level of *AsGLP1* in the roots or nodules in symbiosis process tested by qRT-PCR. All columns were compared to the uninoculated root sample at 1 dpi. The error bars represent the standard deviations of three independent experiments. Significant differences were determined by Student *t* test (*, *P* < 0.05; **, *P* < 0.01). (B to F) Expression patterns of *Mhopa22* promoter-*lacZ* fusion in *A. sinicus* root hairs or nodules. Roots or nodules were observed at 1 dpi (B), 7 dpi (C), 10 dpi (D), 15 dpi (E), and 25 dpi (F) after inoculation with *M. huakuii* 7653R carrying pGD*opa22*. Scale bar, 100 μm.

The relative expression level of *AsGLP1* was measured by quantitative reverse transcription-PCR (qRT-PCR) in total RNA extracted from the roots or nodules of *A. sinicus* under free-living conditions and symbiotic conditions at different days postinoculation (dpi). The results showed that the relative expression level of *AsGLP1* significantly increased at 7 and 10 dpi but gradually decreased after 14 dpi (Fig. 4A), suggesting that highly induced expression of *AsGLP1* is involved in early root nodulation. The relative expression level of *AsGLP1* in early nodules was higher than that in mature nodules, indicating that the transcription of *AsGLP1* in roots is induced by *M. huakuii* 7653R in a symbiotic manner.

Furthermore, the promoter of *Mhopa22* was cloned and fused with the reporter *LacZ* in the construct pGD-*opa22*. The spatial expression pattern of *Mhopa22* in process of symbiosis was detected based on changes in the enzymatic activity of β-galactosidase in the roots and nodules of *A. sinicus* inoculated with *M. huakuii* 7653R harboring pGD-*opa22*. As a result, *Mhopa22* was expressed in infection threads (ITs) (Fig. 4B), young nodules (Fig. 4C), and the infection zone of mature nodules (Fig. 4D to F), implying that *Mhopa22* plays an important role in early rhizobial infection and nodulation. Overall, the expression pattern of *Mhopa22* was consistent with that of *AsGLP1* during symbiosis.

### Effect of *AsGLP1* overexpression on the nodule number of *A. sinicus*.

**FIG 5:**
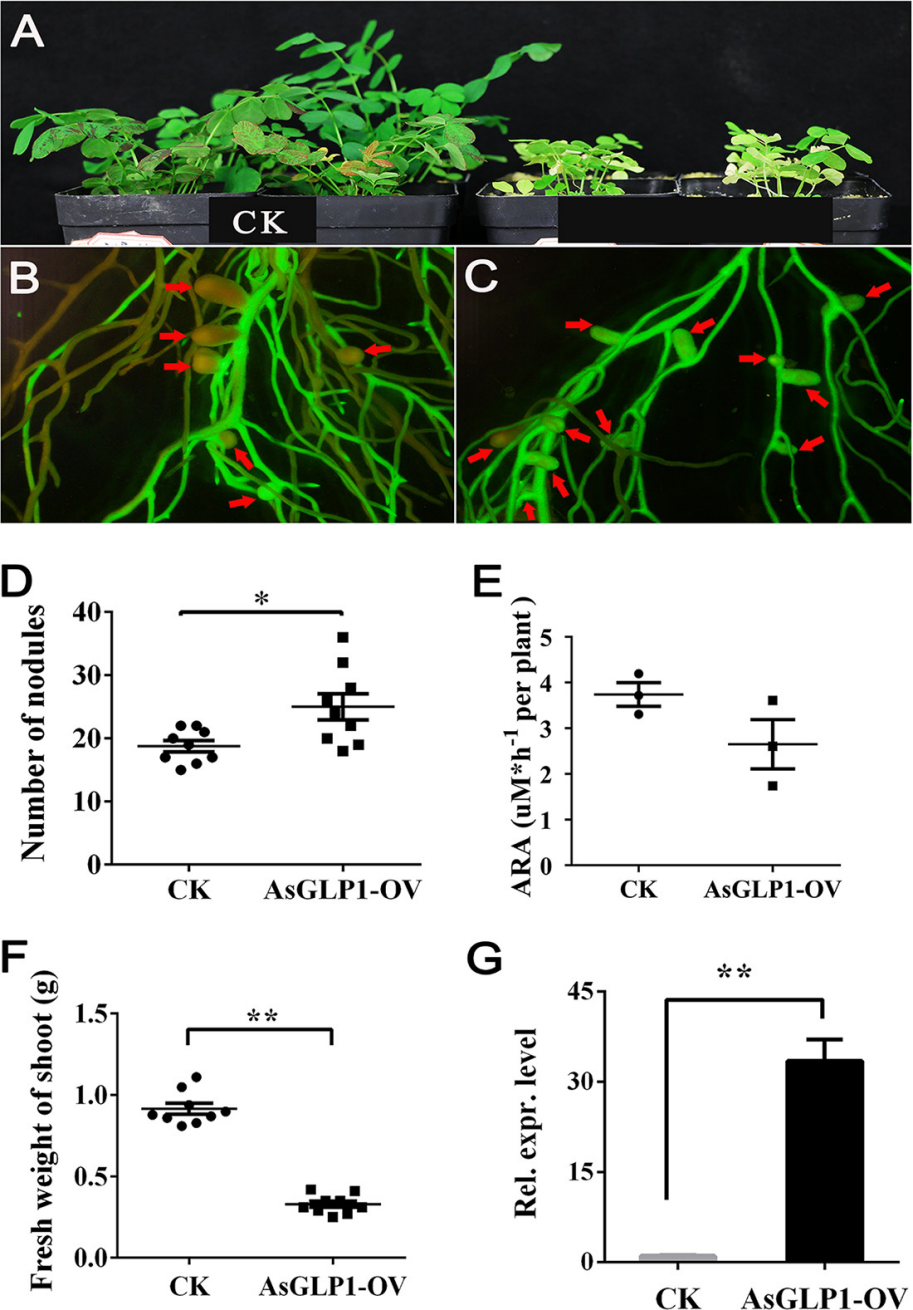
Symbiotic phenotypes of transgenic *A. sinicus* with *AsGLP1* overexpression. (A) Appearance of control (left, CK) and overexpression (right, AsGLP1-OV) plants at 4 weeks. (B and C) Appearance of positive transformation roots with nodules of control and overexpression plants. The nodules are marked with red arrow. (D to F) The number of nodules, acetylene reductase activity and fresh weight of shoot on overexpression and control plants, respectively (*n* = 9). (G) qRT-PCR results showing the efficiency of overexpression in hairy roots. The error bars represent the standard deviations of three independent experiments for panels E and G, and nine independent experiments for panels D and F. Significant differences were determined by Student *t* test (*, *P* < 0.05; **, *P* < 0.01). ARA, acetylene reductase activity; Rel. expr. level, relative transcript level.

The CDS of *AsGLP1* was cloned into the pUB-GFP to obtain the pUB-*AsGLP1*, in which the expression of *AsGLP1* was driven by the *Ljubq1* promoter (44). The pUB-GFP plasmid and its derivatives were also used in other plants, such as *M. truncatula* (45), *G. max* (46), and Oryza sativa (47). At 4 weeks after inoculation with *M. huakuii* 7653R, the results of pot experiments showed that the plants carrying pUB-*AsGLP1* (indicated as *AsGLP1-*OV) formed significantly more nodules (25.00 ± 2.041, *n* = 9) than the control (CK; 18.78 ± 0.8784, *n* = 9) (Fig. 5B to D). However, *AsGLP1-*OV showed yellowish leaves (Fig. 5A), and the fresh weight of the aboveground parts was only 0.33 ± 0.06 (*n* = 9), which was significantly lower than that of CK (0.92 ± 0.11 g) (Fig. 5F). There was no significant difference in acetylene reductase activity of nodules in *AsGLP1-OV* plants relative to that of the CK (Fig. 5E). The expression level of *AsGLP1* in *AsGLP1-OV* plant nodules was detected by qRT-PCR, indicating that *AsGLP1* is successfully overexpressed (Fig. 5G). The results suggested that the overexpression of *AsGLP1* increased the number of nodules, but with a significant decrease in nitrogenase activity due to the abnormal development of nodules.

### Effect of *AsGLP1* RNAi on the nodule number of *A. sinicus*.

**FIG 6:**
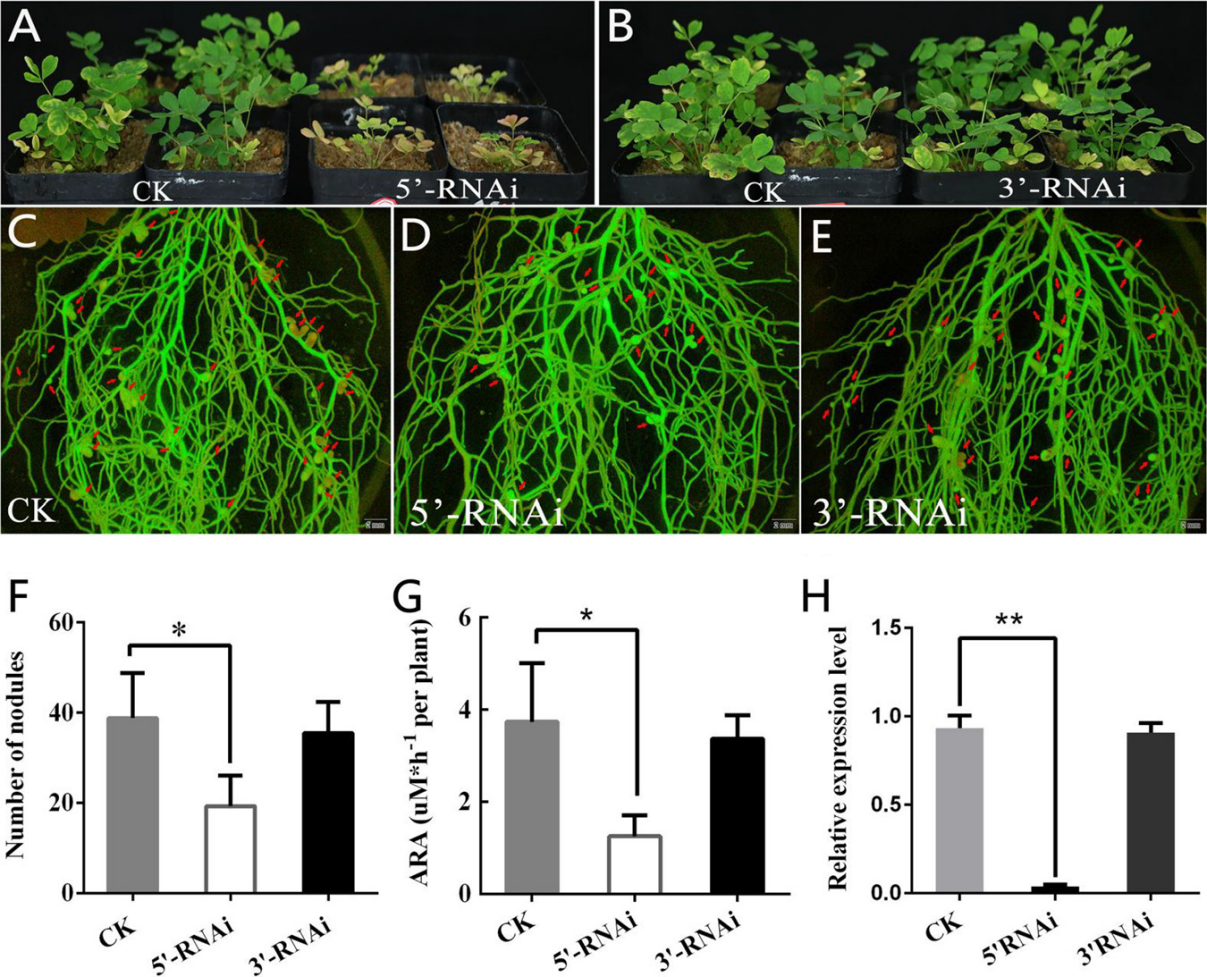
Symbiotic phenotype of *A. sinicus* with *AsGLP1* 5’RNAi or 3’RNAi after 4 weeks of inoculation. (A and B) Appearance of control (CK), *AsGLP1* 5′RNAi (5′RNAi), and *AsGLP1* 3′RNAi (3′RNAi) plants. (C to E) The closeup scope of roots of control (CK), *AsGLP1* 5′RNAi (5′RNAi), and *AsGLP1* 3′RNAi (3′RNAi) plants under a fluorescence microscope (GFP). Scale bar, 2 mm. The nodules are indicated by red arrows. (F to G) Numbers of nodules and acetylene reductase activity per plant. The abbreviation of ARA is short of acetylene reductase activity. (H) qRT-PCR showing the efficiency of RNAi in hairy roots (control and two kinds of RNAi; *n* = 9). The error bars represent the standard deviations of three independent experiments for panel G and nine independent experiments for panels F and H. Significant differences were determined by Student *t* test (*, *P* < 0.05; **, *P* < 0.01).

To identify the symbiotic phenotypes of *AsGLP1*, RNA interference (RNAi) was performed to silence the gene expression of *AsGLP1*. Two target recombinant plasmids pUB-5′-RNAi and pUB-3′-RNAi were constructed and transformed into *A. sinicus* by hairy root transformation, respectively, and an empty plasmid pUB-RNAi was used as the control (CK). The expression of *AsGLP1* in 5′ RNAi exhibited a significant decrease compared to CK, but the 3′ RNAi did not (Fig. 6H). After 4 weeks of growth in the pot, the plants with 5′ *AsGLP1-*RNAi were dwarfed with yellow leaves and small white nodules, whereas the CK plants were vigorous aboveground and had red nodules on the roots (Fig. 6A, C, and D). The growth and nodulation of plants with 3′ *AsGLP1-*RNAi were similar to those of the CK plants (Fig. 6B and E), because the expression of the *AsGLP1* gene was not affected (Fig. 6H). Further quantitative analysis showed that the number of nodules formed on 5′ RNAi plants was 19.3 ± 6.8 (*n* = 9), which was significantly smaller than that for the CK (38.8 ± 9.9, *n* = 9) (Fig. 6F). The acetylene reduction activity, which reflects the nodule nitrogenase activity, was significantly decreased in 5′ RNAi nodules compared to that in CK nodules at the individual plant level (Fig. 6G). These results indicated that the effective silencing of *AsGLP1* impaired root nodule formation.

### Symbiotic phenotype of homologous gene *MtGLPx* in *M. truncatula*.

The results discussed above showed that *AsGLP1* plays an essential role in the process of nodule formation of *A. sinicus*. Hence, it is necessary to characterize the function of its homologous genes in a model legume-rhizobia symbiosis. We conducted further experiments with *M. truncatula* both due to its available genome (48–52) and a large collection of insertion mutants (53). A total of 42 homologous genes were found in *M. truncatula* (https://phytozome.jgi.doe.gov/), which encode the proteins annotated as germin or germin-like proteins, including rhicadhesin receptor. Among these genes, *medtr7g111240.1* had a 79% similarity and the closest phylogenetic relationship with AsGLP1 (see Fig. S2). Therefore, we chose this gene to perform further analysis. We named *medtr7g111240.1* “*MtGLPx*” to distinguish it from *MtGLP1*, another *GLP* annotated by Doll et al. (54).

**FIG 7:**
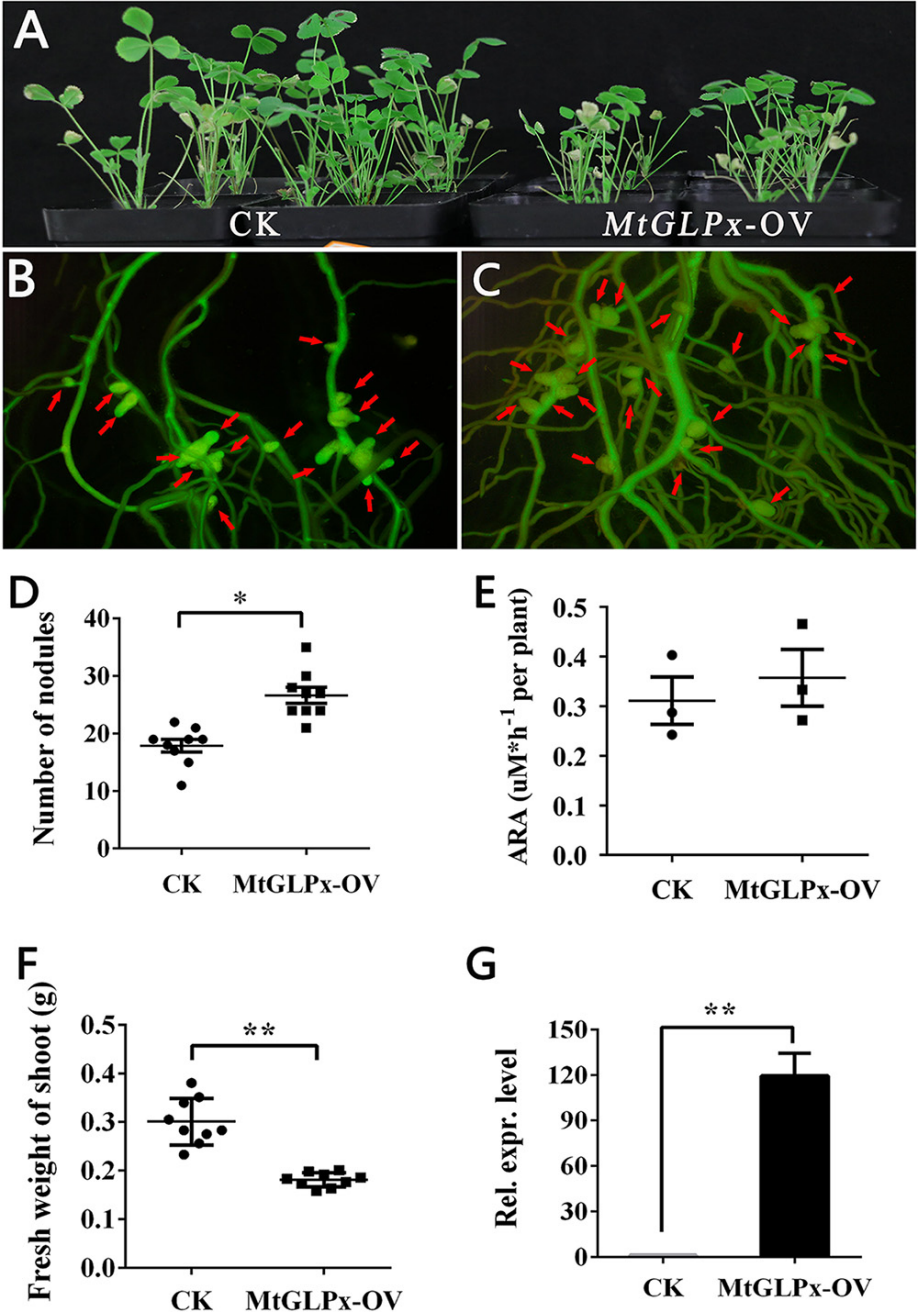
Symbiotic phenotypes of transgenic *M. truncatula* with *MtGLPx* overexpression. (A) Appearance of control (left, CK) and overexpression (right, *MtGLPx*-OV) plants at 4 weeks. (B and C) Appearance of positive transformation roots with nodules of control (B) and overexpression (C) plants. The nodules are marked with red arrow. (D to F) The number of nodules, acetylene reductase activity, and fresh weight of shoot in overexpression and control plants, respectively (*n* = 9). (G) qRT-PCR showing the efficiency of overexpression in hairy roots. The error bars represent the standard deviations of three independent experiments for panels E and G and nine independent experiments for panels D and F. Significant differences were determined by Student *t* test (*, *P* < 0.05; **, *P* < 0.01). ARA, acetylene reductase activity; Rel. expr. level, relative transcript level.

The symbiotic phenotype of *MtGLPx* overexpression was examined in *M. truncatula* transformant mediated by *A. rhizogenes* MUS440 carrying the recombinant plasmid pUB-*MtGLPx* after 4 weeks of inoculation with S. meliloti 1021. Similar results were observed as in *A. sinicus*. The plants with *MtGLPx* overexpression were shorter than the control (CK) (Fig. 7A). The nodule number of *MtGLPx* overexpression plants was 26.7 ± 1.4 (*n* = 9), which was larger than that of the CK (17.9 ± 1.1, *n* = 9) (Fig. 7B to D). The nodule nitrogenase activity in the hairy roots of *MtGLPx* overexpression plants was not significantly different than that of the CK (Fig. 7E). The shoot fresh weight of the plants with *MtGLPx* overexpression was significantly lower than that of the CK (Fig. 7F). The expression level of *MtGLPx* in *MtGLPx-OV* plant nodules was detected by qRT-PCR, indicating that *MtGLPx* is successfully overexpressed (Fig. 7G).

**FIG 8:**
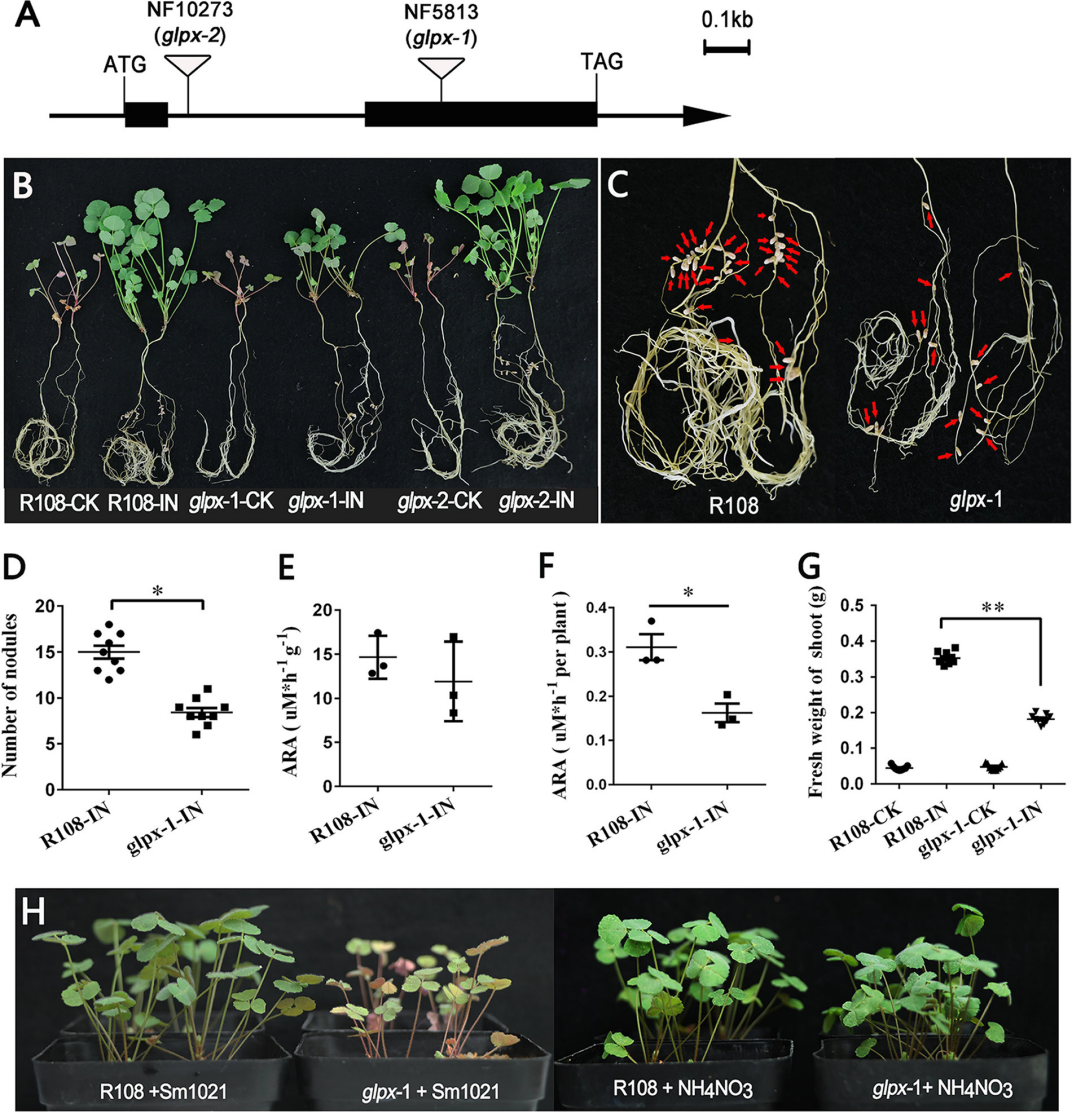
Symbiotic phenotype of *M. truncatula glpx* mutant. (A) Genetic and biochemical characterization of *M. truncatula medtr7g111240.1* is depicted, and the exons are indicated by black boxes. NF5813 (*glpx-1*) and NF10273 (*glpx-2*) mutant lines contained insertion in exon 2 and intron, respectively. (B) Appearance of wild-type R108 and *glpx-1* and *glpx-2* mutant plants inoculated with S. meliloti 1021 (R108-IN, *glpx*-1-IN, and *glpx*-2-IN) or not (R108-CK, *glpx*-1-CK, and *glpx*-2-CK) at 28 dpi. (C) Close-up scope of WT-R108 and *glpx*-1-IN roots, and the nodules are marked with arrows. (D to G) Numbers of nodules, acetylene reductase activity (ARA) per g of nodules, acetylene reductase activity per plant, and fresh weight of shoot (all treatments, *n* = 9), respectively. The error bars represent the standard deviations of three independent experiments for panel E and nine independent experiments for panels of D, F, and G. Significant differences were determined by Student *t* test (*, *P* < 0.05; **, *P* < 0.01). (H) Growth status of 20-day-old *M. truncatula* WT-R108 and mutant *glpx-1* under symbiotic conditions (left) or nonsymbiotic conditions (right) with 5 mM NH_4_NO_3_ added to the nitrogen-free medium.

To further characterize the biological function of *MtGLPx*, we examined the symbiotic phenotypes of Tnt1 insertion mutant of *M. truncatula*. As depicted in Fig. 8A, the genetic and biochemical characterization showed that the *medtr7g111240.1* gene of *M. truncatula* comprised two exons and one intron. Two mutant lines were identified and obtained from Tnt1 insertion mutant seeds provided by the Noble Research Institute (https://medicago-mutant.noble.org/mutant/index.php), namely, NF5813 (*glpx-1*) and NF10273 (*glpx-2*), which contained insertion in exon 2 and the intron, respectively. The symbiotic phenotypes of wild-type *M. truncatula* R108 (WT-R108) and the two mutant lines were assayed after 4 weeks. Under symbiosis, the *glpx-1* exhibited very weak growth, and the *glpx-2* mutant showed growth similar to that of WT-R108 (Fig. 8B). Further quantitative analysis showed that the shoot weight of *glpx-1* was only 0.177 ± 0.023 g (*n* = 9), which was about 50% that of WT-R108 (0.35 ± 0.017 g) (Fig. 8G). The nodule number in *glpx-1* was 8.4 ± 0.5 (*n* = 9), which was significantly smaller than that in WT-R108 (15.0 ± 0.7, *n* = 9) (Fig. 8C, D). The nodules of both *glpx-1* and WT-R108 were red with similar structures, as indicated by the toluidine blue staining of nodule paraffin sections (data not shown), suggesting that the nodules of *glpx-1* were fully filled with bacteroids. Compared to WT-R108, *glpx-1* showed no significant change in nodule nitrogenase activity per unit weight, but the nodule nitrogenase activity for the whole plant showed a significant decrease (Fig. 8E and F). In addition, the nitrogen-deficient growth phenotype of *glpx-1* under symbiosis could be restored by the supply of modified Fahraeus medium with the addition of an extra 5 mM NH_4_NO_3_ (Fig. 8H), indicating that the defective symbiotic phenotype of *glpx-1* with S. meliloti 1021 was due to the deficiency of nitrogen, which was supposed to be fixed by root nodules. Overall, it was revealed that the knockdown of *MtGLPx* resulted in consistent symbiotic phenotypes with the RNAi of *AsGLP1*, suggesting that GLP1 plays an essential role in early nodulation during legume-rhizobium interaction.

### Effect of *glpx-1* disruption on infection.

**FIG 9:**
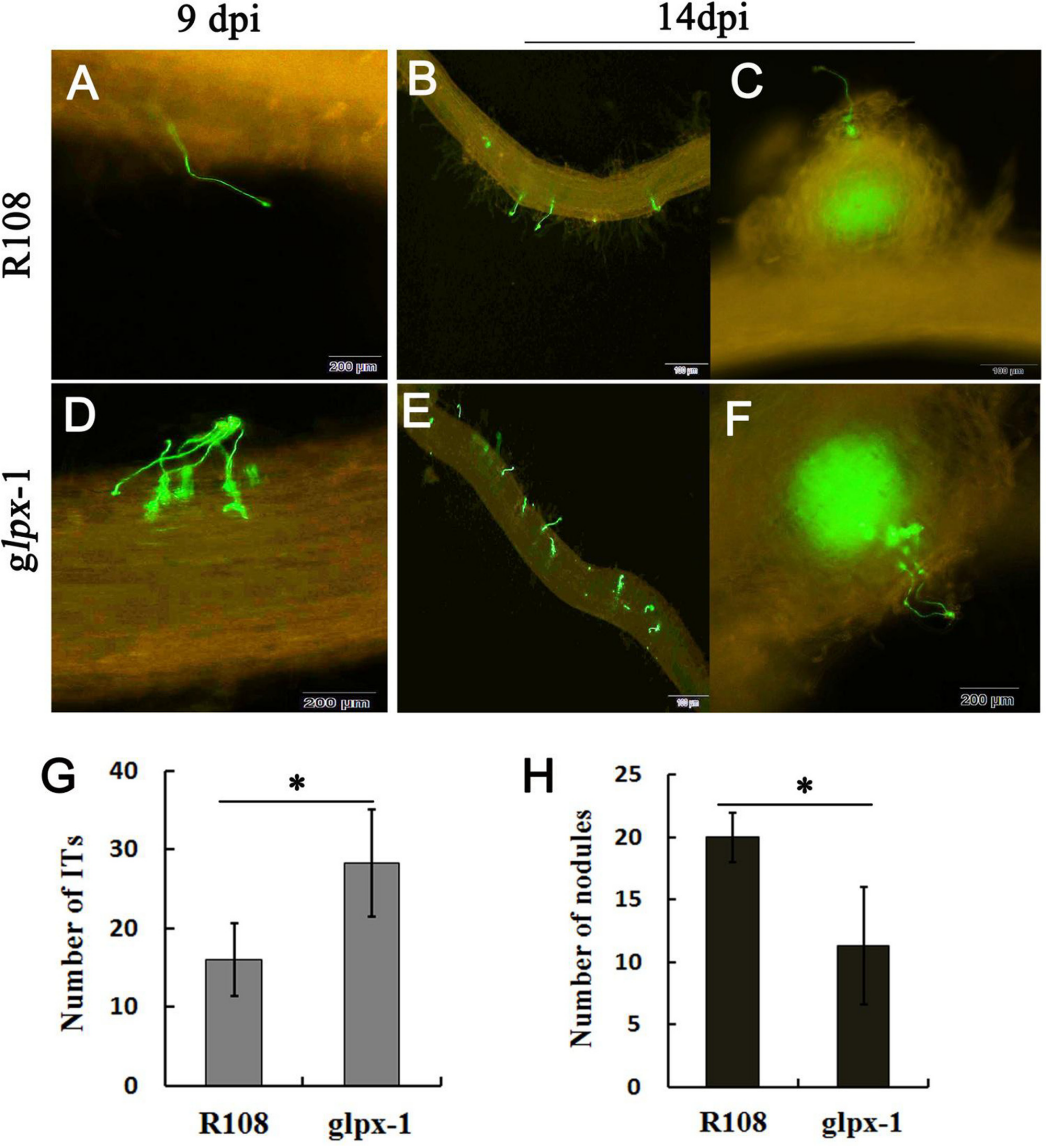
Observation of infection events in *M. truncatula* WT-R108 and *glpx-1* strains inoculated with S. meliloti 1021 marked by GFP. (A to F) Appearance of ITs in WT-R108 and *glpx-1* mutant roots at 9 dpi and 14 dpi. Scale bars, 100 μm (B, C, and E) and 200 μm (A, D, and F). (G) Numbers of infection threads (ITs) in WT-R108 and *glpx-1* strains at 9 dpi (*n* = 3). (H) Appearance of nodules in WT-R108 and *glpx-1* mutant roots at 14 dpi (*n* = 3). The error bars represent the standard deviations of three independent experiments. Significant differences were determined by Student *t* test (*, *P* < 0.05).

To determine which stage of nodule formation is influenced in *glpx-1* mutant, we observed and quantified rhizobial infection events. The roots of *glpx-1* were inoculated with S. meliloti 1021 carrying a green fluorescent protein (GFP) reporter. The results showed that compared to WT-R108, *glpx-1* displayed a significant increase in the number of ITs at 9 dpi (Fig. 9G) but fewer nodules at 14 dpi (Fig. 9H). Notably, a higher intensity of ITs were formed or multiple ITs occurred on one site of nodule primordium in *glpx-1* roots (Fig. 9A to F). This observation indicated that the disruption of *MtGLPx* would result in a nearly 2-fold increase in the number of ITs, which is usually considered an abnormal phenomenon in nodule formation and development. It is believed that hyperinfection is due to the disruption of balance between symbiosis and defense (55, 56), which leads to a decrease in root nodule number. Taken together, these findings show that *MtGLPx* plays an important role in the formation and development of ITs and/or nodule primordium.

### Coordinated changes in the expression of symbiotic and defensive genes in the *glpx-1* mutant.

**FIG 10:**
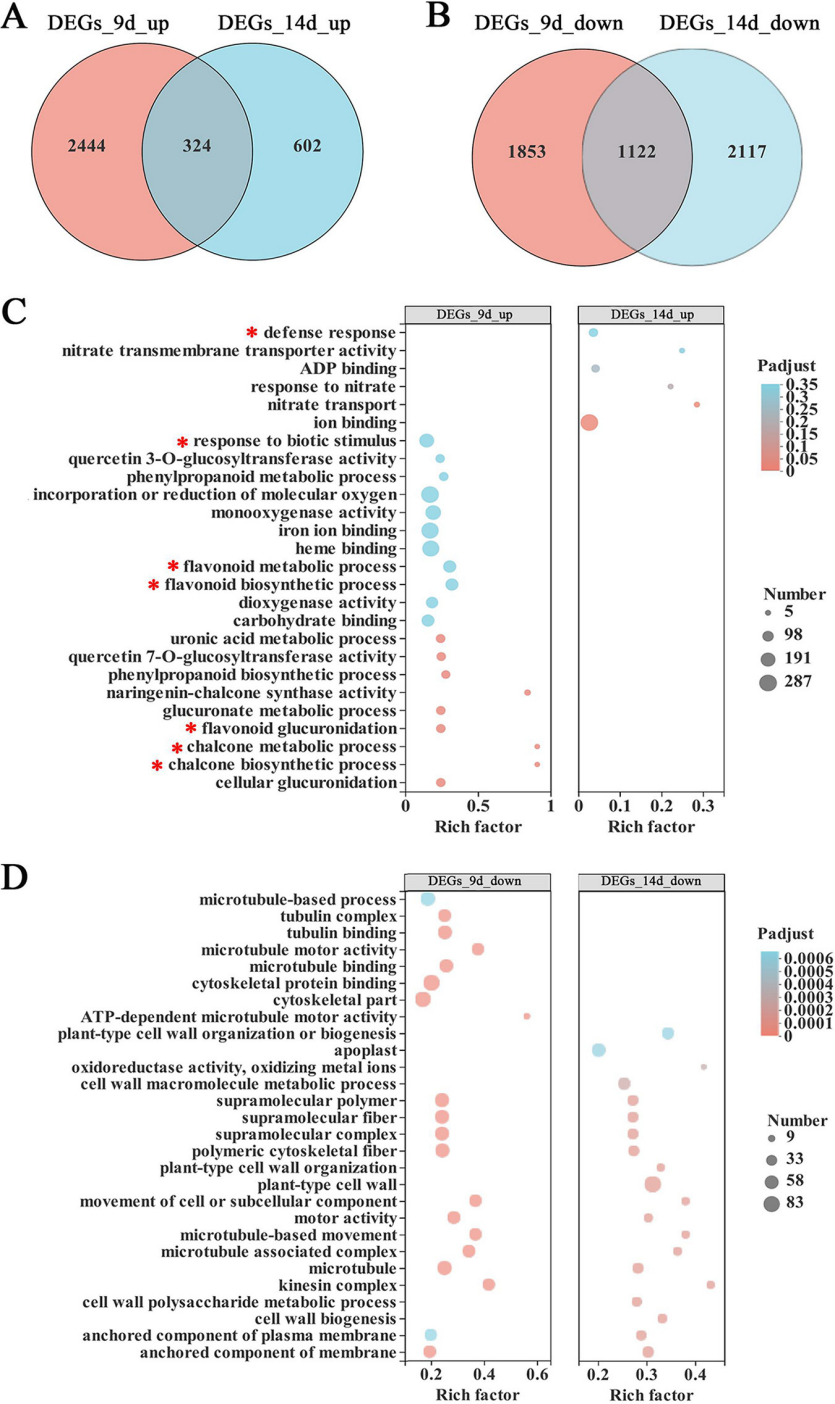
Gene expression profile in *Mtglpx-1* mutant at the early stage of nodulation. (A and B) Venn diagram of DEGs between *glpx-1*_9d and R108_9d and between *glpx-1*_14d and R108_14d, respectively. (C and D) GO enrichment analysis of all significantly upregulated and downregulated *Mtglpx-1* genes compared to those in WT-R108 at 9 and 14 dpi, respectively. The abscissa represents rich factor, and the ordinate represents GO term/KEGG pathway. The sizes of the dots represent the numbers of genes/transcripts in the GO term, and the colors of the dots correspond to the different adjusted *P* (P_adjust_) value ranges. The red asterisk marks the term/KEGG pathway related to symbiotic and defensive genes.

In order to investigate the potential functional mechanism of *MtGLPx*, a global transcriptome analysis of roots for WT-R108 and *glpx-1* at 9 and 14 dpi was performed by RNA-seq (the time points were chosen due to the large difference in IT number between WT-R108 and *glpx-1* during this symbiosis period). The data showed that there were 5,743 differentially expressed genes (DEGs) between *glpx-1*_9d and R108 9d (2,768 up- and 2,975 downregulated) and 4,165 DEGs between *glpx-1*_14d and R108_14d (926 up- and 3,239 downregulated), indicating the distinct gene expression between WT-R108 and *Mtglpx* at these time points. Among these DEGs, only 324 genes were upregulated and 1,122 genes were downregulated at both time points (Fig. 10A and B). The GO enrichment analysis revealed that many genes related to the flavonoid metabolic process were upregulated at 9 dpi (marked by a red asterisk in Fig. 10C), but they were no longer differentially expressed at 14 dpi, and instead, some genes associated with defense response and ion binding were upregulated. However, the classification of downregulated DEGs at both 9 and 14 dpi seemed be independent of symbiosis and defense process (Fig. 10D). Some special DEGs at 9 and 14 dpi between the wild type and mutant are listed in Tables S2 and S3, respectively.

More specifically, various hormone genes displayed different tendencies. For example, the genes responsive to auxin (auxin efflux carrier family transporter), gibberellin (gibberellin 2-β-dioxygenase), and ethylene (ethylene-responsive transcription factor) were downregulated and the abscisic acid receptor and jasmonate zim-domain protein were upregulated. On the other hand, the chalcone synthase, flavonol synthase, and Nod factor-binding lectin-nucleotide phosphohydrolase were upregulated, but no obvious change was found for the expression of nodulin genes (see Table S2). These results implied that the loss of function of *MtGLPx* would disturb the hormone response and influence the coordinated expression of symbiotic and defensive genes. The variations in quantity and category of DEGs indicated the relationship between the *Mtglpx* function and the regulation of symbiotic signaling pathway.

## DISCUSSION

The recognition between plants and compatible rhizobia upon their physical contact is regarded as a crucial step for the initiation of the symbiotic pathway (57). Great progress has been achieved in elucidating the symbiotic mechanism, particularly the Nod factor-dependent signaling pathway, over the past decade (58). Here, we identified a germin-like protein AsGLP1 from *A. sinicus*, which interacts with an OMP *Mhopa22* of rhizobia. Germin and germin-like proteins are generally thought to have redox-type enzymatic activities and not be involved in binding to bacterial partners (59). One exception might be the rhicadhesin receptor (43). This study is the first report a germin-like protein in legumes that mediates symbiotic nodulation by interacting with the OMPs of rhizobia.

It has been reported that the extracellular loops of partial OMPs within human-pathogenic bacteria are hypervariable and responsible for the association with the receptor in the host, such as HV1 (HyperVariable Loop 1) and HV2 (HyperVariable Loop 2) of Opa60 from *Neisseria* that are the critical regions for binding with CEACAMs (carcinoembryonic antigen-like cellular adhesion molecules) on human cells and initiating the engulfment of the bacterium (60, 61). Similarly, OmpA2 Loop 4 from Porphyromonas gingivalis plays a crucial role in the formation of biofilm, which facilitates the bacterial invasion to the host (62). Structurally, the Mhopa22 protein is predicted to form a typical β-barrel possessing four extracellular loops (see Fig. S3), which could provide a basis for the interaction with the host plant.

AsGLP1 identified in this study, and its orthologs are annotated as rhicadhesin receptor in the NCBI database. Rhicadhesin, a surface-located protein in *Agrobacterium* and Rhizobium leguminosarum, was supposed to facilitate the association of bacteria with the plant cell wall (43) and mediate the first step of direct attachment (63). Exogenous addition of crude rhicadhesin to live rhizobia would diminish their attachment to plant cell wall (64, 65). However, it remains unclear what encodes rhicadhesin and how its putative activity is related to the attachment, and the specific gene has not been cloned (66). Our results suggest an important role for the interaction between AsGLP1 and Mhopa22. Further experiments are needed to determine whether Mhopa22 is rhicadhesin.

Here, we found that the carboxyl-terminal end of AsGLP1, which contains the two germin boxes, is an essential region that mediates its interaction with Mhopa22 (Fig. 3). KGE was found to be present within the germin boxes 2 (from K138 to E140) (Fig. 1A), and the tripeptide KGD or KGE was characterized in almost 50% of GLPs (67, 68), but their functions are not determined yet. In mammalian cells, these tripeptides might be responsible for extracellular functions and intracellular protein-protein interactions (69, 70). Therefore, the tripeptide KGE of AsGLP1 may be the critical amino acid residue involved in its interaction with Mhopa22. Also, our findings provide new insights into the symbiotic molecular interaction between rhizobia and the host plant.

It has long been regarded that GLPs belong to germin superfamily with the conserved cupin domain and beta-barrel structure (71). They are derived from different plants and can be divided into different clusters (72). There are 21 germin-like genes in soybean (GmGER1 to -21) (67) and at least 40 in *M. truncatula*. However, there have been very few studies to characterize the role of GLPs in the process of symbiosis. The earliest study revealed that a cell wall-located GLP identified from pea facilitates the attachment of *Rhizobium* and *Agrobacterium* bacteria to the plant cell wall by associating with the bacterial protein rhicadhesin (43). Gucciardo identified a germin-like protein (PsGER1) from Pisum sativum that showed protein sequence identity with the N-terminal sequence of a putative plant receptor for rhicadhesin, and it was speculated that PsGER1 is required for the first stage of bacterial attachment to root hairs based on the specific localization of PsGER1 mRNA in the nodule apex (73). However, there has been no further report of the molecular mechanism for the symbiotic interaction.

Functionally, we demonstrated that the RNAi of *AsGLP1* resulted in a decrease in nodule number, whereas *AsGLP1* overexpression increased the number of nodules in the hairy roots of *A. sinicus*. Similarly, the knockdown of *MtGLPx* in *M. truncatula* also resulted in a decrease in nodule number. The symbiotic phenotypes of *AsGLP1* RNAi and *MtGLPx* mutants are different from those of *Mhopa22* mutant, which do not form nodules and do not seem to form infection threads on *A. sinicus*. We think that the difference for *A. sinicus* may be due to the fact that RNAi did not completely eradicate *AsGLP1* expression. In the case of *M. truncatula*, the system may be different enough that nodule formation and IT formation are not completely dependent on the interaction between *MtGLPx* and the S. meliloti version of *Mhopa22*. Interestingly, an abnormal infection event was observed in the roots of *glpx-1* inoculated with S. meliloti 1021, which was defined as hyperinfection. This phenomenon is usually associated with abnormal nodule development as described in a few legume mutants (55, 56). These results imply that the loss of function of *glpx* in the mutant disrupts the balance between symbiosis and defense reactions during the nodulation. The RNA-seq results could provide some clues to support the phenotype of *M. truncatula* mutant *glpx-1* as discussed below.

Time-course transcriptomic experiments of *M. truncatula* supported the hypothesis that during rhizobia-legume symbiosis, defense response of plant must be suppressed or evaded by rhizobia to successfully reach the nodule through ITs and eventually be intracellularly accommodated (74). Balanced regulation of innate immunity is required throughout rhizobial infection, symbiosis establishment, and maintenance (18, 75). RNA-seq data showed that a number of genes associated with phytohormone displayed changes of expression in mutant roots or nodules at 9 and 14 dpi (see Tables S2 and S3). The expression of defense genes was remarkably increased at 9 dpi (Fig. 10C). The stronger defensive stress for the steady invasion of rhizobia could be partial reason for the decrease in nodule number despite the formation of more ITs. Importantly, many hormone genes displayed changes in expression. For example, the genes responsive to auxin (auxin efflux carrier family transporter), gibberellin (gibberellin 2-β-dioxygenase), and ethylene (ethylene-responsive transcription factor) were downregulated, whereas the genes associated with the abscisic acid receptor and jasmonate zim-domain protein were upregulated. Mounting studies demonstrated that the phytohormone homeostasis regulates the mutualistic symbiotic interaction between legumes and rhizobia by controlling the formation of ITs (76). Auxin is regulated by NF signaling and required in nodule meristem for cortical division (77, 78). The gibberellin involved in Nod factor triggered negative feedback (79), the ethylene downregulates early genes of symbiotic pathway (80), and abscisic acid negatively regulates ENOD11 and RIP1 (81). In our data, the disturbed expression of the genes related to defense and phytohormone homeostasis implied the imbalanced coordination of symbiosis and defense signaling pathways in *MtGLPx* mutation. Therefore, it can be concluded that *MtGLPx* plays an essential role in the development of ITs and nodule primordia during early symbiosis.

In summary, based on the interaction between Mhopa22 and AsGLP1 and the functional characterization, we speculate that GLP1 plays an essential role in mediating early symbiotic process through interacting with the OMPs of rhizobia. Our findings provide novel insights into the molecular mechanism of rhizobia-legume interaction during nodulation, as well as a new perspective for further studying the elements and pathway in the recognition between rhizobial OMPs and legume plants in the future.

## MATERIALS AND METHODS

### Plant growth, inoculation, and bacterial strain.

Wild-type *A. sinicu*s collected from Xinyang, China, was used for phenotype analysis, as well as for the overexpression or RNAi of *AsGLP1*. The seeds were germinated on the medium with 0.5% sucrose and 1.2% agar after surface sterilization by 75% ethanol for 5 min and 2% sodium hypochlorite for 10 min (82). Wild-type (WT) *M. truncatula* R108 was used for *MtGLPx* overexpression or RNAi and used together with two homozygous *glpx* mutants (*glpx-1* and *glpx-2*) isolated from the Noble Research Institute *M. truncatula* mutant collection by a PCR-based reverse screening approach for phenotype analysis and expression profiling. The sterilization and germination of *M. truncatula* seeds were performed as described previously (83). The germinated seeds were transferred to pots filled with sterile sand in the chamber for 16 h at 24°C/8 h at 18°C, day/night cycle with 40 to 60% humidity and irrigated with Nitrogen-free Fahraeus Solution (NFS). After 5 to 7 days, the plants were inoculated with appropriate rhizobia, i.e., *A. sinicus* with *M. huakuii* 7653R and *M. truncatula* with S. meliloti 1021. The phenotypic analysis was conducted after 4 weeks of inoculation. Both *M. huakuii* 7653R and S. meliloti 1021 were cultured in TY medium containing 50 μg L^−1^ streptomycin at 28°C. Agrobacterium rhizogenes K599, *A. rhizogenes* MSU440, and A. tumefaciens EHA105 were used for hairy root transformation in *A. sinicus* and *Medicago* spp. and for transient expression in Nicotiana benthamiana, respectively. Wild-type tobacco plants were grown in a growth chamber at 22°C and 70% relative humidity under a 16-h-light/8-h-dark photoperiod for approximately 6 weeks.

### Fragment cloning and vector construction.

For BiFC, the full-length sequence of *AsGLP1* was obtained by PCR with the primers pairs BI-1X/BI-213X and then subcloned into pSCYCE-R at the site of XbaI and XhoI, resulting in recombinant plasmid, including pSPYNE(R)-AsGLP1(1-213). The *Mhopa22* gene was reamplified by PCR (BI-opaB/BI-opaX) and inserted between the BamHI and XhoI sites of pSCYNE to obtain *Mhopa22*::SCFPN. The *in planta* expression of all fusion proteins in these constructs was under the control of 35S promoter.

Similarly, for yeast two-hybrid assay, the Mhopa22 coding sequence was amplified using the primer YH-opaE/YH-opaP and inserted into the pGBKT7 at the site of EcoRI and PstI to obtain the bait plasmid pGBKT7-*Mhopa22*. For further identification of the binding site, the four truncated fragments that encode AsGLP1(19-213), AsGLP1(58-213), AsGLP1(122-213), and AsGLP1(1-130) were obtained by PCR with the primers YH-19E/YH-213X, YH-58E/YH-213X, YH-122E/YH-213X, and YH-1E/YH-130X, respectively, and then inserted into pGADT7 to obtain four constructs: pGADT7-AsGLP1(19-213), pGADT7-AsGLP1(58-213), pGADT7-AsGLP1(122-213), and pGADT7-AsGLP1(1-130).

For overexpression, the primers OV-AsX/OV-AsS and OV-MtX/OV-MtS were used to amplify the ORF of *AsGLP1* and *MtGLPx*, respectively, and the products were inserted between the XbaI and SacI sites of pUB-GFP to obtain pUB-*AsGLP1* and -*MtGLPx*. The RNAi plasmid was derived from pUB-GFP. Briefly, the primers pUBRni-F (CCAGTGCCAAGCTGGGCTGCAACTAGTTCCCCAGATTAGCCTTTTCAATTTC) and pUBRni-R (TATGACCATGATTACGAATTCGAGCTAGATCTCCAAGCTTGATGCATGTTGTCAATCAA) were used to amplify a 1,746-bp fragment from pCAMBIA1301-35S-int-T7 and then fused with SacI and PstI digested pUB-GFP to obtain the pUB-RNAi. Two *AsGLP1*-RNAi constructs were used: 5′ RNAi contained a 174-bp coding sequence 8 bp downstream of the first codon (Met1), which was amplified using the primer Rni5′-(cis)S/Rni5′-(cis)B or Rni5′-(anti)P/Rni5′-(anti)X, while 3′ RNAi encompassed a 43-bp coding sequence and a 189-bp 3′ untranslated region amplified using the primer Rni3′-(cis)S/Rni3′-(cis)B or Rni3′-(anti)P/Rni3′-(anti)X. The forward primers contained SacI-BamHI sites, and the reverse primers had PstI-XbaI sites. The fragments of *AsGLP1* were cloned into pUB-RNAi in inverse orientations with an *actin* intron between them. The RNAi constructs were expressed under the control of the CaMV35S promoter (84).

To construct the pGD-opa plasmid, a 610-bp PCR fragment containing the *Mhopa22* promoter region was amplified using the Pro-opaH and Pro-opaB primers, digested with BamHI and HindIII, and cloned into the in-frame orientation at the same site of the *lacZ* translational fusion plasmid pGD926 (56).

To assay the interaction between Mhopa22 with AsGLP1 *in vitro*, the nucleotide fragment coding AsGLP1(58-213) was amplified by PCR using PD-58(His)E/PD-213(His)X and was inserted between the EcoRI and XhoI sites of pET-28a(+) (Novagen, Germany) to allow the expression of an His-AsGLP1(58-213) fusion protein. The Mhopa22 expression construct was designed without the N-terminal signal sequence, i.e., residues 1 to 17 were replaced with Met1. The coding sequence of Mhopa22(J) was amplified using PD-opaJE/PD-opaJX primers, digested by EcoRI and XbaI, and then inserted into the pMAL-c2x vector (New England Biolabs, Ipswich, MA), resulting in the generation of the recombinant plasmid to express MBP-Mhopa22(J) fusion proteins in the E. coli Rosetta(DE3) (Novagen). All constructs—together with primers, restriction enzyme cleavage sites, and brief descriptions—are listed in Table S4.

### Bimolecular fluorescence complementation assay.

A bimolecular fluorescence complementation (BiFC) assay was performed by referring to the method described by Kang et al. (85). A. tumefaciens GV3101 cells containing different paired plasmids (different combinations of pSPYCE and pSPYNE-R or their derivatives) were cultured in Luria-Bertani (LB) liquid medium, collected by centrifugation, resuspended in infiltration buffer, and then mixed, followed by the adjustment to an optical density at 600 nm (OD_600_) of 0.5; they were then used to transform the A. tumefaciens GV3101 (pMP90) strains, respectively. After incubation for 2 to 4 h at room temperature, the A. tumefaciens GV3101 mixture was injected into the leaves of 6-week-old Nicotiana benthamiana plants. Subsequently, the fluorescence was assayed at 3 to 5 days after infiltration using a laser-scanning microscope with cyan fluorescent protein (CFP; excitation wavelength, 405 nm; emission wavelength, 477 nm).

### Protein-protein interaction in yeast cells.

In order to capture the interacting protein, S. cerevisiae Y187 harboring pGBKT7-*Mhopa22* mixed with the *A. sinicus* AD-cDNA library containing the total RNA isolated from the root and nodule tissues of *A. sinicus* at different developmental stages infected by *M. huakuii* 7653R was used (86). For further identification of the binding site of AsGLP1 with Mhopa22, the yeast S. cerevisiae AH109 carrying pGADT7 and four derivatives (to express the truncated AsGLP1) were hybridized with Y187 (pGBKT7-*Mhopa22*), and the fusion yeast was dropped on the DDO and QDO/X-Gal. The operation was performed according to the methods described in the Clontech yeast protocols handbook (84). The interaction between mammalian p53 and SV40 served as a positive control, whereas that of lamin (Lam) with SV40 served as a negative control. The method of β-galactosidase assay was as previously described (84).

### *In vitro* MBP pulldown assay.

The pilot experiment results showed that the maltose-binding protein (MBP)-Mhopa22(J) fusion proteins were expressed in E. coli Rosetta after induction with 1 mmol L^−1^ IPTG for 12 h at 20°C and were partially soluble, and the His-AsGLP1(58-213) fusion protein existed in the form of inclusion body. Hence, the purification of target product was conducted via degeneration-renaturation as previously described (87). After induction by 1 mmol L^−1^ isopropyl β-d-1-thiogalactopyranoside (IPTG) at 37°C for 4 h, cells from 250 mL culture were harvested by centrifugation (5,000 × *g*, 20 min, 12°C), resuspended in 15 mL of lysis buffer (50 mmol L^−1^ Tris-HCl [pH 8.0], 300 mmol L^−1^ NaCl, 1 mol L^−1^ phenylmethanesulfonylfluoride), and lysed using a microfluidizer. The insoluble fraction was washed by precipitate washing buffer (10 mmol L^−1^ Tris-HCl [pH 8.0], 300 mmol L^−1^ NaCl, 1 mol L^−1^ EDTA, 1% Triton, and 1 mol L^−1^ Urea) and then pelleted again. The fusion protein was solubilized from inclusion bodies in 15 mL Denaturation-Renaturation (DR) buffer (50 mmol L^−1^ Tris-HCl [pH = 7.8], 5 mmol L^−1^ MgCl_2_) containing 6 M urea incubated at 4°C for 1 h. The mixture was centrifuged at 10,000 × *g* for 20 min at 4°C, the supernatant was dialyzed gradually against DR buffer containing progressively lower concentrations of urea (4, 2, 1, and 0 mol L^−1^) overnight, and finally, the supernatant was obtained after centrifugation at 10,000 × *g* for 20 min at 4°C.

The pulldown assay was conducted as previously described (88) with minor modifications. Briefly, the MBP-Mhopa22(J) fusion protein was purified and bound to the MBP-Affinity Resin (New England Biolabs, catalog no. E8021V). MBP beads alone or bound with MBP-tag were used as the negative control. The purified His-AsGLP1(58-213) fusion protein was incubated with the immobilized MBP-Mhopa22(J) fusion protein or the control samples in 1 mL of interaction buffer (20 mmol L^−1^ Tris-HCl, 100 mmol L^−1^ KCl, 2 mmol L^−1^ MgCl_2_, 5% glycerol [pH 8.0]) for 1 h at 4°C with shaking. After reaction, the beads were washed for three times with phosphate-buffered saline (PBS; 2.7 mmol L^−1^ KCl, 140 mmol L^−1^ NaCl, 1.8 mmol L^−1^ KH_2_PO_4_, 10 mmol L^−1^, Na_2_HPO_4_ [pH 8.0]) containing 1% Triton X-100, followed by three washes in PBS. The protein complexes were separated via SDS-PAGE and His-AsGLP1(58-213) was detected with anti-His tag antibody.

### Observation of infection events.

To detect the temporal and spatial expression patterns of *Mhopa22*, the roots of *A. sinicu*s inoculated with *M. huakuii* 7653R carrying pGD-opa were collected at different time points (1, 7, 10, 15, and 25 dpi), incubated in *LacZ* staining solution [0.1 mol L^−1^ phosphate buffer (pH 7.4), 0.08% X-Gal, 5 mmol L^−1^ K_3_Fe(CN)_6_, and 5 mmol L^−1^ K_4_Fe(CN)_6_] and stained overnight at 28°C, followed by fixation with 2% (vol/vol) glutaraldehyde solution for 0.5 h under vacuum and three rinses with 0.1 mol L^−1^ phosphate buffer. Entire roots or nodule sections were observed under a light microscope, and under the stereomicroscope after *lacZ* dyeing.

For observation of infection events in mutant plants, wild-type *M. truncatula* and *glpx* mutant were inoculated with S. meliloti 1021 harboring pMP2463, which carried the eGFP report gene derived from the *LacZ* promoter (89). The roots were collected at different time points (3, 7, 9, and 14 dpi) and observed under a fluorescence microscope.

### Hairy root transformation.

The *A. rhizogenes* mediated *A. sinicus* transformation was carried out as described previously (82) with minor modifications. Briefly, the *A. rhizogenes* K599 line containing overexpression plasmids (pUB-*AsGLP1* and empty pUB-GFP) or RNAi plasmids (empty pUB-RNAi, pUB-5′RNAi and pUB-3′RNAi) were cultured in 50 mL of LB medium until the OD_600_ reached ~1.0. Sterilized 5-day-old seedlings were cut in the middle of the hypocotyl and incubated in bacterial suspension culture for 10 min. After blotting with autoclaved filter paper, the seedlings were placed on Murashige and Skoog basal medium and grown under 16-h/8-h light/darkness at 24°C/20°C, respectively. Three days later, the explants were transferred to fresh Murashige and Skoog medium with 500 mg L^−1^ carbenicillin (Duchefa) and 30 mg L^−1^ kanamycin (Duchefa) and grown for 10 more days until hairy roots developed from hypocotyls. The plants harboring positive transgenic hairy roots identified by using GFP were transferred to pots for phenotype analysis. For *M. truncatula*, the transfection was performed as described previously (83). The method for hairy root transformation of *M. truncatula* differed from that of *A. sinicus*. In order to obtain the transformed *M. truncatula* containing the pUB-*MtGLPx*, the operational process was performed according to the protocol of *M. truncatula* handbook (90).

### mRNA sequencing analysis.

To investigate the influence of *MtGLPx* deficiency in *M. truncatula*, global gene expression profiles in WT-R108 and *glpx-1* nodule-stripped roots at 9 and 14 dpi were assessed by mRNA sequencing (RNA-seq; the transcriptome analysis was assisted by Majorbio). Identified genes with significant upregulation and downregulation were mapped (fold change ≥ 2 and *P < *0.05). Based on GO annotations, the changes in genes associated with symbiosis, defense mechanism, and molecular functions were analyzed. All the analyses were based on the integrated cloud platform of I-Sanger (https://cloud.majorbio.com/).

### qRT-PCR.

Temporal RNA expression patterns of *AsGLP1* from *A. sinicus* were analyzed in the extracts from roots and nodules at different developmental stages (1, 3, 7, 10, 14, 21, and 28 dpi). Total RNA was extracted from each sample using TRIzol reagent (Invitrogen, USA) according to the manufacturer’s protocol. DNase I (TaKaRa, Japan) was used to remove genomic DNA. RNA purity was assessed by calculating the OD_260_/OD_280_ ratio of each sample. An aliquot of 3 μg of total RNA was used for reverse transcription. First-strand cDNA was synthesized by using RevertAid reverse transcriptase (Fermentas, USA) using oligo(dT)_18_ primers. qRT-PCR was performed with the SYBR Premix ExTaqII (TaKaRa, Japan). The data were analyzed by the 2^–ΔΔ^*^CT^* method (91) with *AsActin* as the reference gene (92). Each experiment was performed in triplicate. One-way analysis of variance (ANOVA) with the Duncan test (*P* ≤ 0.05) was applied in SPSS v.20 (IBM Corp., New York, NY) to compare differential expression values between different developmental stages of nodules and tissues.

### Nitrogenase activity assay.

Nitrogenase activity was assessed by the acetylene reduction activity method (93). For each sample, nine root lines were analyzed. Every three hypogeal parts of hairy root plants (including nodules and roots) were incubated in 2 mL of acetylene for 2 h at 28°C in 20-mL glass bottles with rubber seals. The amount of ethylene was measured using an East & West Analytical Instrument GC 4000A gas chromatograph.

### Statistical analysis.

The significance of the data was analyzed using an independent-sample Student *t* test, and multiple comparison was performed using one-way ANOVA method by SPSS 15.0. A *P* value of ≤0.05 was considered statistically significant. Data were represented as means ± the standard errors of the mean. Bars in the figures represent the standard errors of three independent experiments.

### Data availability.

The cDNA sequence of AsGLPl has been submitted to GenBank of NCBI under accession number MZ669228. Raw RNA-seq data were submitted to the NCBI BioProject database under BioProject accession number PRJNA905920.
