# Supplementary material for: A Germin-Like Protein GLP1 of Legumes Mediates Symbiotic Nodulation by Interacting with an Outer Membrane Protein of Rhizobia
Source: Microbiol Spectr. 2023 Jan 12;11(1):e03350-22. doi: 10.1128/spectrum.03350-22 (PMC9927233; doi:10.1128/spectrum.03350-22)
Supplement: Supplemental file 1 — Supplemental material. Download spectrum.03350-22-s0001.pdf, PDF file, 0.9 MB [file spectrum.03350-22-s0001.pdf]

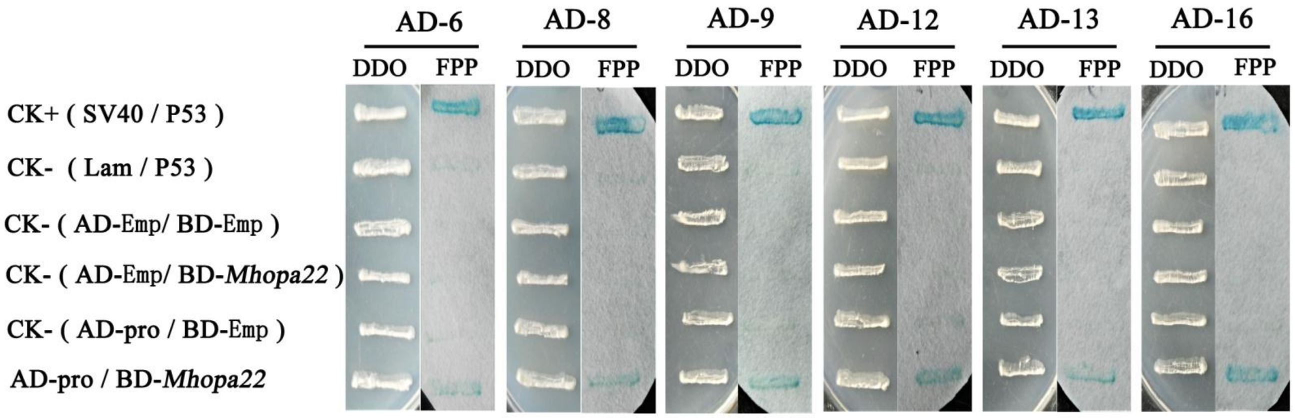

FIG S1 Interaction of Mhopa22 with six candidate proteins from *Astragalus sinicus* in a Y2H assay by the filter paper photocopy. Yeast was transformed with bait and prey constructs. The β-galactosidase assay [5-bromo-4-chloro-3-indolyl-β-d-galactopyran-oside (X-gal)] was performed on a filter paper print (FPP) of DO-Leu-Trp (DDO) grown yeast. SV40/P53 interaction was used as a positive control, and SV40/P53, AD-Emp/BD-Emp, AD-Emp/BD-*Mhopa22* and AD-pro/BD-Emp interactions were used as negative controls. Among them, AD-Emp, BD-Emp and BD-*Mhopa22* refer to the empty vector pGADT7, the empty vector pGBKT7 and pGBKT7-*Mhopa22*, respectively.

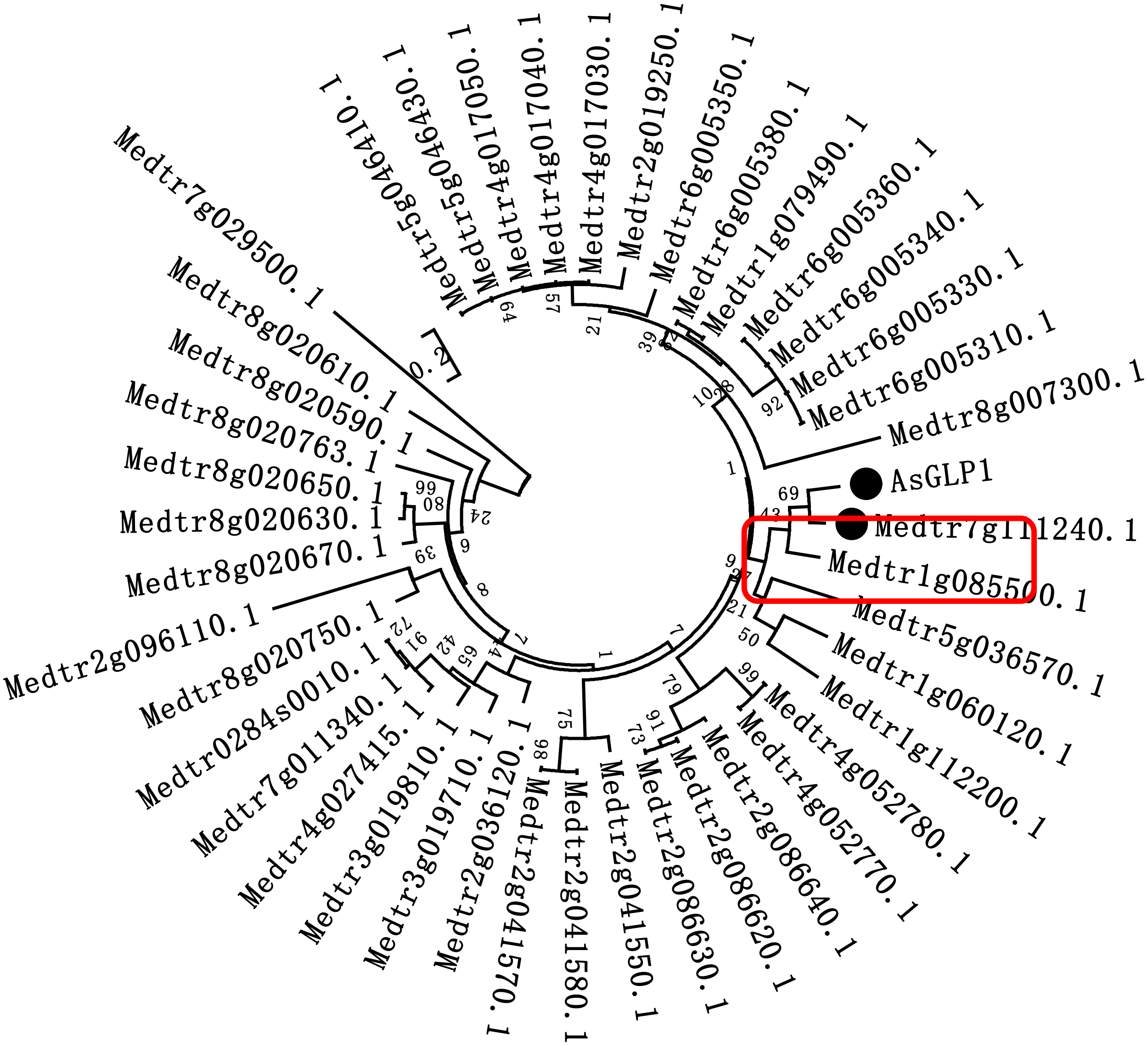

FIG S2 Phylogenetic analysis of AsGLP1 and GLPs in *Medicago truncatula* based on amino acid sequences. A total of 42 genes in the genome of *M. truncatula* were searched on the website https://phytozome.jgi.doe.gov/ by using the description words *germin* or *germin-like protein.* The phylogenetic tree was constructed by neighbor-joining analysis in MEGA 6. medtr7g111240.1 has an amino acid sequence (213 residues) sharing 79% similarity and the closest relationship to AsGLP1 (shown in red box).

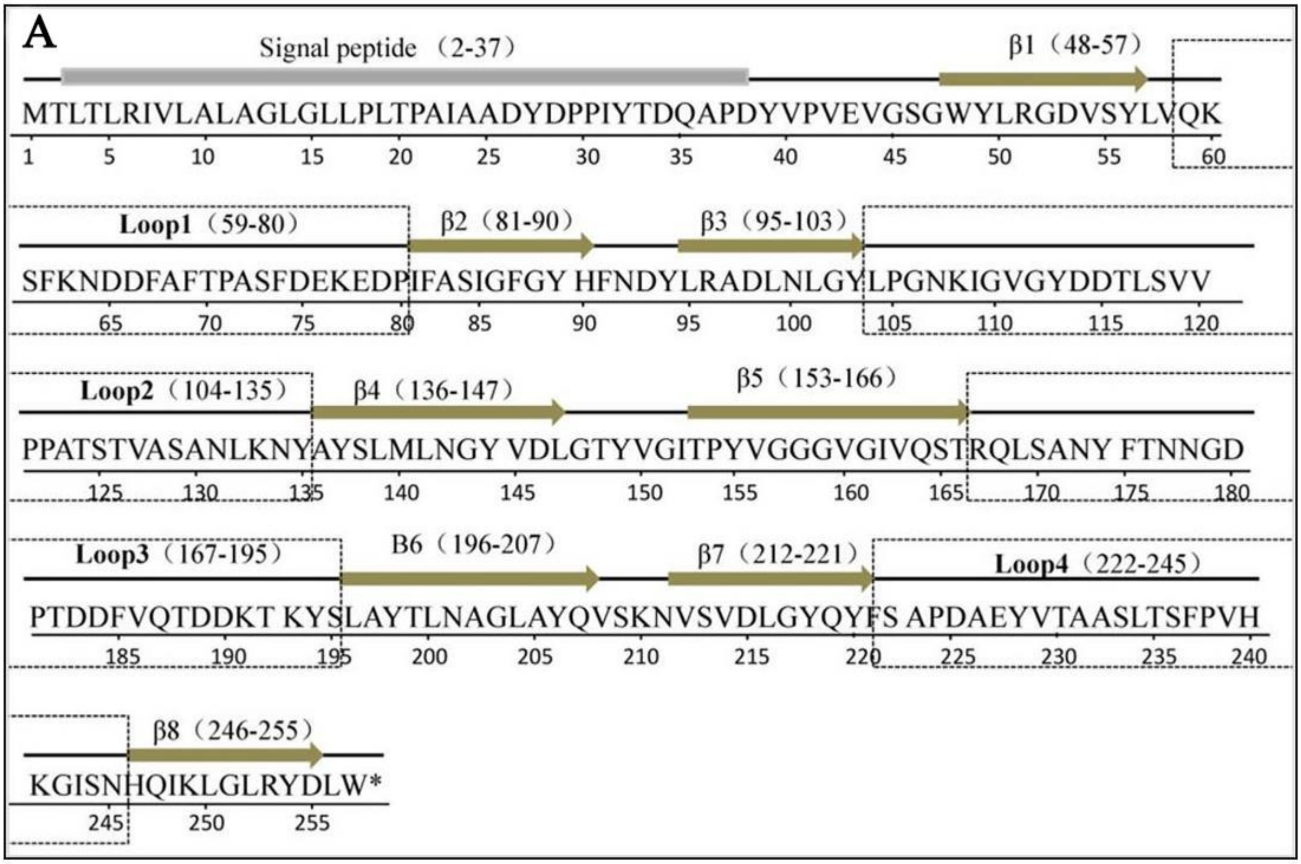

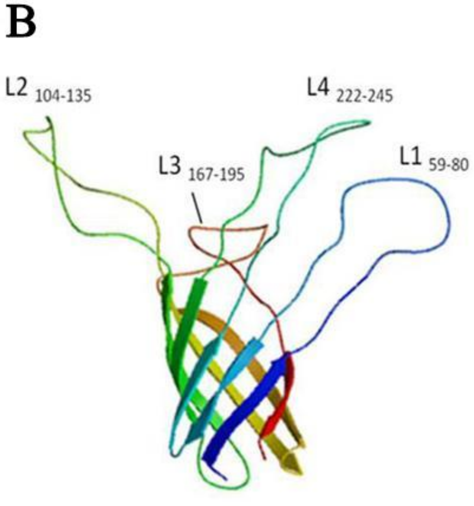

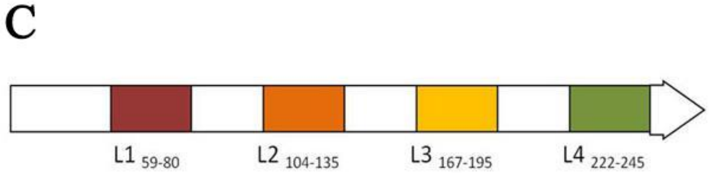

D

Peptide	Sequence
L1	QKSFKNDDFAFTPASFDEKEDP
L2	LPGNKIGVGYDDTLSVVPPATSTVASANLKNY
L3	RQLSANYFTNNGDPTDDFVQTDDKTKYS
L4	SAPDAEYVTAASLTSFPVHKGISN

FIG S3 In silico analysis of Mhopa22 protein. (A) Sequence and structure of Mhopa22. The primary peptide sequence and secondary structure were obtained from the PDBsum database. (B) Structure modeling of Mhopa22, which displays transmembrane β-barrel and predicted extracellular loops L1-L4. (C) Schematic representation of the location of the extracellular loops (color corresponding to β-barrel image) in Mhopa22. (D) Display of predicted extracellular loop sequences of Mhopa22. L1-L4 represent the Loop1-Loop4, respectively.

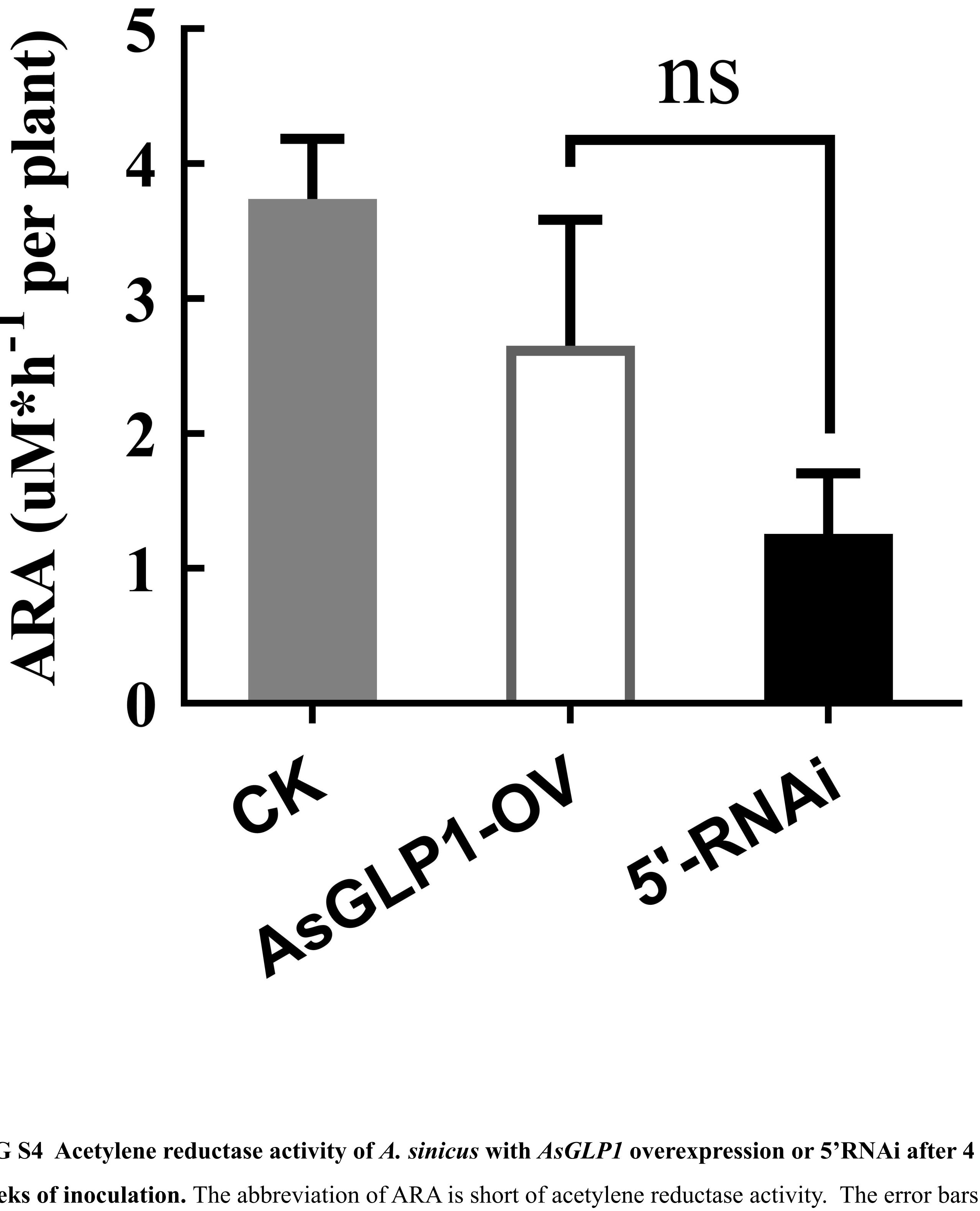

FIG S4 Acetylene reductase activity of *A. sinicus* with *AsGLP1* overexpression or 5'RNAi after 4 weeks of inoculation. The abbreviation of ARA is short of acetylene reductase activity. The error bars represent the standard deviations of three independent experiments. "ns" indicated no significant differences by Student's *t* test.

TABLE S1 Aligned results of BLASTp

No.	Top BLAST hit	The number of amino acids encoded by fragments
AD-6	Cysteine proteinase	85
AD-8	NADH dehydrogenase	153
AD-9	Coiled-coil-helix-coiled-coil-helix domain-containing protein 10	171
AD-12	NADH dehydrogenase 1 beta Subcomplex subunit 3-B	87
AD-13	Rhicadhesin receptor	81
AD-16	Hypothesis proterin	69

By using the Mhopa22 (carryed in pGBKT7) as bait protein fishing for prey encoded by *A. sinicus* cDNA library with the system of yeast two-hybrid (Y2H). Interactions were selected on dropout (DO) medium lacking Leu (L), His (H), adenine (A), and Trp (W), supplemented with 5 mm 3-AT. Six DNA fragments on pGADT7 (AD for short) were selected from fifty-seven blue fusion after the elimination of repeat sequences and frameshift mutations due to the sequence inserted not in the frame with Gal4 activation domain. The proteins coded by these sequences predicted in NCBI listed in this table.

TABLE S2 Part of differentially expressed genes in *glpx* mutant at 9 dpi

Gene name	FC (*glpx*_9d/ R108_9d)	Gene description
**Hormone-related gene**		
MTR_4g084870	0.312	auxin efflux carrier family transporter
MTR_6g488150	0.36	auxin response factor
MTR_1g034060	0.002	gibberellin 2-beta-dioxygenase
MTR_2g043050	0.106	ethylene-responsive transcription factor ERF017
MTR_5g083270	2.262	abscisic acid receptor
MTR_5g013530	19.733	jasmonate zim-domain protein
**Defense -related gene**		
MTR_2g043150	32.553	Defensin fusion
MTR_8g012775	21.681	Defensin-like protein
MTR_8g090305	35.193	disease resistance protein (NBS-LRR class)
MTR_8g014930	14.045	LRR receptor-like kinase
**Symbiosis-related gene**		
MTR_5g007717	10.764	chalcone and stilbene synthase family protein
MTR_5g007723	10.998	chalcone synthase
MTR_1g115850	3.087	chalcone-flavanone isomerase family protein
MTR_3g025250	0.197	flavonoid hydroxylase
MTR_1g027290	31.62	flavonol synthase/flavanone 3-hydroxylase
MTR_4g073230	104.698	receptor-like kinase
MTR_5g081030	0.377	leghemoglobin Lb120-1
MTR_0115s0030	0.298	Nodule Cysteine-Rich (NCR) secreted peptide
MTR_3g055440	0.498	nodulin-25 protein
MTR_4g082860	0.34	nodulin-like protein
MTR_1g090957	551.572	legume lectin beta domain protein
MTR_7g085200	7.991	Nod factor-binding lectin-nucleotide phosphohydrolase
MTR_8g006120	21.116	nodulin MtN21/EamA-like transporter family protein
MTR_4g094555	8.358	stress up-regulated Nod 19 protein
MTR_4g113820	2.964	early nodulin 93

FC (*glpx*_9d/R108_9d) represents the fold change of *Mtglpx*_9d compared with R108_9d.

TABLE S3 Part of differentially expressed genes in *glpx* mutant at 14 dpi

Gene name	FC (*glpx*_14d/ R108_14d)	Gene description
**Hormone-related gene**		
MTR_4g127100	0.08	auxin efflux carrier family transporter
MTR_8g020610	0.148	auxin-binding protein ABP19a
MTR_4g063130	0.384	auxin-regulated protein
MTR_4g102670	0.039	ethylene response factor
MTR_2g043020	0.167	ethylene-responsive transcription factor ERF017
MTR_4g100450	2.645	ethylene response factor
MTR_7g010580	0.054	gibberellin-regulated family protein
MTR_1g086550	0.093	plant gibberellin 2-oxidase
**Defense -related gene**		
MTR_6g074865	2.232	defensin
MTR_7g095970	4.339	defensin-like protein
MTR_8g010290	2.325	defensin-like protein
MTR_8g010270	5.683	defensin-like protein
MTR_5g027860	2.219	disease resistance protein (CC-NBS-LRR class)
MTR_4g081280	14.465	disease resistance protein (TIR-NBS-LRR class)
MTR_5g070470	2.393	disease resistance protein RGA3
MTR_1g056370	4.391	disease resistance response protein
MTR_3g032110	16.531	LRR and NB-ARC domain disease resistance protein
MTR_6g046450	2.361	NB-ARC domain disease resistance protein
**Symbiosis-related gene**		
MTR_1g076940	3.211	isoflavone-7-O-methyltransferase
MTR_5g081030	2.014	leghemoglobin Lb120-1
MTR_8g078300	12.398	Nod-factor receptor 5, putative
MTR_3g436100	0.488	leginsulin related MtN11/16/17 family
MTR_5g085790	0.283	LysM-domain receptor-like kinase
MTR_1g038560	0.08	nodulin MtN21/EamA-like transporter family protein
MTR_4g082860	0.317	nodulin-like protein
MTR_4g113820	0.352	early nodulin 93

FC (*glpx*_14d/R108_14d) represents the fold change of *Mtglpx*_14d compared with R108_14d.

TABLE S4 Primers used in this work

Gene	Primer name	Sequences (5'→3')
Mhopa22	YH-opaE	CGGAATTCATGACACTCACATTGCGTATTGT
Mhopa22	YH-opaP	GCCTGCAGGCTACCAGAGATCGTAGCGCAGT
Mhopa22	BI-opaB	CGGGATCCATGACACTCACATTGCGTATTGT
Mhopa22	BI-opaX	CCGCTCGAGCTACCAGAGATCGTAGCGCAGT
Mhopa22	Pro-opaH	CCCAAGCTTGCGCGTCAAGTGGGATACCG
Mhopa22	Pro-opaB	CGGGATCCAGCACAATACGCAATGTGAGT
Mhopa22	PD-opaJE	CGGAATTCATGACACTCACATTGCGTATTGT
Mhopa22	PD-opaJX	GCTCTAGATTACCAGAGATCGTAGCGCAGT
AsGLP1	YH-1E	CGGAATTCATGAAGCTAGTAGCAGTGTTGTTTTTTGTGG
AsGLP1	YH-213X	CCGCTCGAGTCACTTAGGTGCAAGCCTAGCCTTAAT
AsGLP1	YH-19E	CGGAATTCGATCCTGGTGCTCTTCAAGACC
AsGLP1	YH-58E	CGGAATTCCTAGCAAAAGCAGGTGCAACAAAC
AsGLP1	YH-122E	CGGAATTCGTTGGTTTTATTACTACAGCAAATGTG
AsGLP1	YH-130X	CCGCTCGAGTCACACATTTGCTGTAGTAATAAAACCAAC
AsGLP1	BI-1X	GCTCTAGAATGAAGCTAGTAGCAGTGTTGTTTTTTGTGG
AsGLP1	BI-213X	GCCTCGAGCTTCTTAGGTGCAAGCCTAGCCTTAAT
AsGLP1	BI-58X	GCTCTAGAATGCTAGCAAAAGCAGGTGCAACAAAC
AsGLP1	BI-130X	GCCTCGAGCACATTTGCTGTAGTAATAAAACCAAC
AsGLP1	OV-AsX	GCTCTAGAATGAAGCTAGTAGCAGTGTTGTTTTTTGTGG
AsGLP1	OV-AsS	GCGAGCTCCTACTTCTTAGGTGCAAGCCTAGCCTTAAT
AsGLP1	Rni5'-(cis)S	GCGAGCTCAGCAGTGTTGTTTTTTGTGGTTTTG
AsGLP1	Rni5'-(cis)B	CGGGATCCCCTGCTTTTGCTAGTGCGTTTG
AsGLP1	Rni5'-(anti)P	GCCTGCAGAGCAGTGTTGTTTTTTGTGGTTTTG
AsGLP1	Rni5'-(anti)X	GCTCTAGACCTGCTTTTGCTAGTGCGTTTG
AsGLP1	Rni3'-(cis)S	GCGAGCTCGGAGGTTCAGAAAATTAAGGCTAGG
AsGLP1	Rni3'-(cis)B	CGGGATCCACCCCCATTTGTTTTTATTCCTTAT
AsGLP1	Rni3'-(anti)P	GCCTGCAGGGAGGTTCAGAAAATTAAGGCTAGG
AsGLP1	Rni3'-(anti)X	GCTCTAGAACCCCCATTTGTTTTTATTCCTTAT
AsGLP1	PD-58(His)E	CGGAATTCCTAGCCAAACCAGGAGCCAC
AsGLP1	PD-213(His)X	CCGCTCGAGTCACTTAGGTGCAAGCCTAGCCTTAAT
AsGLP1	RT-AsF	TGGATTCGTTTGCAAAGATCCTGCTC

AsGLP1	RT-AsR	GCACGTGGGTGAATATGTGGTGGGT
AsActin	RT-AsActin-F	GTTCTTTTCCAGCCTTCTATGA
AsActin	RT-AsActin-R	ATGTTTCCGTACAGATCCTTTC
MtGLPx	OV-MtX	GCTCTAGAATGAAGATCATCGCAGTGTTGCTG
MtGLPx	OV-MtS	GCGAGCTCCTACTTGGGTGCAAGCCTAGACTTG
MtGLPx	RT-MtF	CCGCAGCTGATTTCTCCACCAACG
MtGLPx	RT-MtR	ACGTGGGTGAGTGTGAGGTGGGTTC
MtEF1-αq	RT-MtEF1F	ACTGGTGGTTTTGAAGCTGGT
MtEF1-αq	RT-MtEF1R	TGGTGGACCTCTCAATCATGT
MtJAD1	JAD-RT-F	GCCAACATGCAAAACTCAGATGGGG
MtJAD1	JAD-RT-R	GGTGCACTTGGGGGGTTTTCTTCG
MtERN1	MtERN1-RT-F	GGAAGATGGTGCTGTTGCTT
MtERN1	MtERN1-RT-F	TGTTGGATTGTGAACCTGACTC
MtNIN	MtNIN-RT-F	GGTCGCCGGTCGTCTTCT
MtNIN	MtNIN-RT-R	GCCTTGGTTCTTCGCTTGTC
